# Experiences of current practice, priorities and strategies for enabling meaningful consumer and community involvement collaborations in health and medical research in Queensland, Australia

**DOI:** 10.1186/s40900-026-00847-y

**Published:** 2026-02-04

**Authors:** Lisa Anemaat, Kimberley A. Baxter, Emmah Doig, Jennifer Muller, Nadine E. Foster, Jessica Schults, Karina R. Charles, Adrienne Young, Silvia Manzanero, Tanya Smyth, Michael O’Sullivan, Gail Robinson, Rachel Latimore, Diana Tam, David A. Copland

**Affiliations:** 1 Surgical Treatment and Rehabilitation Service (STARS) Education and Research Alliance, The University of Queensland and Metro North Health, 296 Herston Road, Herston, Brisbane, QLD 4029 Australia; 2 Queensland Aphasia Research Centre, School of Health and Rehabilitation Sciences, The University of Queensland, Brisbane, Australia; 3 School of Health and Rehabilitation Sciences, The University of Queensland, Brisbane, Australia; 4 School of Nursing, Midwifery and Social Work, Centre for Clinical Research, The University of Queensland, School of Nursing, Midwifery and Social Work, Brisbane, Australia; 5 Herston Infectious Diseases Institute (HeIDI), Brisbane, Australia; 6 Royal Brisbane and Women’s Hospital, Metro North Health, Brisbane, Australia; 7 Centre for Health Services Research, The University of Queensland, Brisbane, Australia; 8 Jamieson Trauma Institute (JTI), Metro North Health, Brisbane, Australia; 9 Metro North Health, Metro North Engage, Brisbane, Australia; 10 School of Medicine, Queensland University of Technology, Brisbane, Australia; 11 School of Pharmacy and Medical Sciences, Griffith University, Brisbane, Australia

**Keywords:** Consumer and community involvement, Consumer partnership, Patient and public involvement

## Abstract

**Background:**

Consumer and community involvement (CCI) in health and medical research is essential for ensuring meaningful and transferrable research. However, CCI practices vary across study types, research fields, and settings, limiting capacity to determine changes needed to enhance partnerships. This study aimed to (1) describe and evaluate current CCI practices; (2) explore experiences; and (3) collaboratively identify priorities and strategies to increase meaningful practice.

**Methods:**

Sequential mixed-methods design, comprising an online cross-sectional survey and in-person priority-setting workshop. Researchers and consumers with experience of CCI relative to health and medical research were eligible. Snowball sampling was used to recruit from six research institutes (4-health-service centres; 2-university centres) and two research-active public hospitals from one health precinct in Queensland, Australia. Four-part cross-sectional survey included: 1-participant characteristics; 2-research characteristics; 3-experiences and priority generation; 4-ranking training and organisational development needs. The survey used multiple-choice, Likert-scales and open-ended questions. Following this, a facilitated priority-setting workshop was undertaken with a sample of consumer and researcher respondents, to identify priorities and strategies to promote meaningful CCI in research. Qualitative analysis and descriptive statistics were applied to survey data. Triangulation was ensured through confirmation of themes across experience data and prioritisation rankings. Data were collected between September 2024 and May 2025.

**Results:**

Survey participants: researchers (*n* = 38) and consumers (*n* = 16). Priority-setting workshop: researchers (*n* = 7) and consumers (*n* = 8). Experiences described CCI across all research cycle stages, and for consumers with varying levels of involvement in decision making in individual studies. For consumers, challenging experiences were often associated with having insufficient input or influence on the research. Challenges for researchers were commonly related to resourcing constraints, limited time for process modifications or communication support, navigating inflexible processes and systems, or finding, approaching and engaging consumers for relevant projects at appropriate times. Six top priorities and strategies for improvement were collaboratively agreed. The top priority called for increased organisational development (e.g., dedicated CCI expertise available for researchers to access).

**Conclusions:**

Research demonstrated increasing application of CCI across research fields. Consumers reported genuine and meaningful involvement but sought clearer role definitions. Implementing identified priorities may result in more adequately resourced CCI in research.

**Supplementary Information:**

The online version contains supplementary material available at 10.1186/s40900-026-00847-y.

## Abstract (plain language summary)

**Background:**

Consumer and community involvement in research can improve care for patients, the management of healthcare services, and result in services that better meet the needs of individuals. However, how consumers are involved varies considerably across different research projects. This research aimed to provide an understanding of where consumer and community involvement in healthcare research is taking place and to identify the resources and training most needed to do this well.

**Methods:**

Researchers and consumers who have previously worked together on a research project were involved. They participated in an online survey to describe their experiences and suggest ideas for change. Similar ideas for improvement that had been described were grouped together to find common themes, and an in-person priority-setting workshop was held to agree on the order of importance of these suggestions.

**Results:**

Participants in the online survey were 38 researchers and 16 consumers, and 7 researchers and 8 consumers attended the priority-setting workshop. Research data was collected between September 2024 and May 2025. Participants were recruited from two hospitals and six research centres in Queensland, Australia. They described experiences taking place across all stages of research, using different research methods, and in different healthcare fields. Together, we agreed on six top priorities for improving how consumer and community involvement in research is undertaken.

**Conclusions:**

Consumer and community involvement is increasing across all stages of research. Top priorities highlighted the importance of developing strategies that help consumers and researchers work effectively together.

**Supplementary Information:**

The online version contains supplementary material available at 10.1186/s40900-026-00847-y.

## Introduction

Insights from lived experience, where patients and community members partner with researchers in the design, conduct and/or evaluation of research, can help research be more relevant, transferable, and able to meet community needs [1–3]. Consumer and community involvement (CCI) also known as patient and public involvement (PPI) in research, encompasses consumer investigator roles, including as members of the research team and advisory roles, involved in making key decisions that direct the research, where lived experience shapes research at any stage [4]. In Australia, the term consumer refers to a person who has lived experience of a disease, disorder, or use of a health service and their family or carers [5]. In this paper, CCI in research refers to the collaborative generation of knowledge between consumers who can be patients, families, carers or community groups and researchers [6], and does not include research participation [7]. The benefits of CCI in research are now well established in the literature [8–10] and supported by policymakers [11, 12] and researchers [13]. In response, CCI is increasingly becoming a requirement of national health standards [11, 12], health service boards [14], and research funders [5, 15, 16] to improve person-centred care and democratise research processes [17, 18]. In addition, there are ethical imperatives, i.e. conducting research ‘*with and by*’, rather than ‘*to*,* about*,* or for*’, to meet the needs and priorities of those most affected [19]. These policy, funding and ethical motivations drive many researchers to work with consumers. The democratising value of CCI in research may additionally enhance the methodological quality of research [20] through increased representativeness of outcomes, recruitment and retention of target populations, and in the co-construction of clinical knowledge [8, 18]. While the benefits of CCI in research have been firmly established, and funding and policy directives have driven an uptake in practice, strategies for ensuring experiences are meaningful and positive for the consumers who partner are less understood.

Over the last decade, uptake in CCI has increased considerably in many areas of health and medical research [21], bringing greater diversity to how researchers partner with consumers across research fields, methods, stages of the research cycle, and levels of involvement. For example, a 2019 systematic review of international CCI practices specific to cancer research found that over the preceding 10 years, CCI had steadily grown, with most studies reporting CCI focused on single, earlier prioritisation stages, and others reporting CCI practices across multiple stages of the research cycle [18]. Another 2019 review of the extent of CCI in health service planning and design research, captured CCI practices from 18 studies, across diverse therapeutic areas including stroke, mental health, drug treatment, HIV services and methodological approaches case study, qualitative descriptive, ethnographic, or grounded theory [22]. Studies included in this review demonstrated broad levels of CCI from collection of feedback to higher-level two-way consumer partnerships [22]. The review also highlighted the complexity of defining meaningful consumer partnership that tailor’s involvement to support consumers by meeting their needs supporting different therapeutic or condition-specific, age-related, cultural, literacy, informational, or location-specific needs [22]. In Australia, the scope of CCI in health research often remains limited to pockets of good practice within individual research groups or focuses on health service governance rather than health research [21, 23–28]. A recent national survey of CCI in Australian clinical trials which included respondents from a wide range of therapeutic areas, found instances of CCI practices reported across multiple research stages [29], however, practices were most commonly limited to reviewing new proposals [29]. Despite the clear benefits of CCI, and availability of over 65 frameworks guiding best practice [30], CCI in research largely remains inadequately formalised [29, 31], and reports of tokenistic or unfulfilling experiences, that lack meaningful involvement for consumers remain [32, 33].

Optimal approaches to facilitate high-quality, collaborative, and meaningful CCI in health research are a growing focus of research efforts [4, 21, 22, 34–37]. Beyond involvement across research cycle stages, the value or meaningfulness of CCI in research can also be considered based on the degree of decision making a consumer holds. For example, the International Association for Public Participation (IAP2) [38], defines a spectrum of decision making across five levels of involvement: inform, consult, involve, collaborate, and empower. These range from being a one-way recipient of knowledge at the inform stage, towards the empower stage where there are increased levels of two-way dialogue and influence on decision-making. Black et al. [35], explored what constitutes meaningful involvement for consumers, by capturing experiences of CCI in research and offering recommendations for improving involvement in the Canadian research context. Study recommendations related to creating a welcoming research environment, managing expectations, and valuing consumer contributions. However, the study did not collect data on research field or design of the relevant studies [35]. Given the heterogeneous nature of research with its broad research fields of basic science, clinical, health services, public health research and the varying methodological approaches and research cycle stages a deeper understanding of what constitutes meaningful consumer involvement is needed that is tailored to each project. In addition, the diversity in needs and preferences of consumers and different population groups means that capturing the perspective of diverse groups of consumers is essential. As CCI in research activities expands across health and medical research, an evaluation of CCI practices is timely to understand current CCI practice, and to identify priorities and strategies to achieve implementation of high-quality, best-practice, meaningful CCI in research. To date, evaluations of CCI in research exploring current processes, practices, and experiences across the stages of the research cycle and levels of CCI influence remain limited.

Therefore, this research aimed to (1) describe and evaluate current CCI in research practices in health and medical research; (2) explore experiences of researchers and consumers; and (3) collaboratively identify priorities and strategies to increase meaningful CCI in research within a metropolitan health precinct in Queensland, Australia.

## Methods

### Study design

A sequential mixed-methods design was used to align with the study aims by first describing and evaluating current CCI practices and experiences through an online cross-sectional survey, followed by an in-person workshop to collaboratively refine and prioritise strategies for strengthening meaningful CCI. The sequential structure ensured survey findings directly informed consensus-based priority setting. The study is part of a broader program of research that included an online survey, interviews, and a collaborative priority-setting workshop. This paper reports the findings from the online survey and priority-setting workshop; interview data will be reported elsewhere. Reporting was guided by the Guidance for Reporting Involvement of Patients and the Public (GRIPP2) long form checklist [16] for reporting patient and public involvement in research.

### Setting

The study was conducted across two large research active metropolitan hospitals in Queensland, Australia that employ more than 9900 multi-disciplinary staff combined, and care for > 600,000 patients annually; four hospital-based research centres specialising in cancer, bio-fabrication, trauma care, and infectious diseases research; and two university-funded hospital-based research centres specialising in aphasia, and recovery from motor vehicle accident research. During the recruitment period, there were a combined total of ~ 1000 active research studies across facilities, including all project cycle stages, from establishment phase to nearing completion.

### Participants and recruitment

Consumers and researchers with experience of CCI in health and medical research were recruited. CCI in research included consumer contributions across one or more research studies, research committees, or as advisors for a research centre or program of research. Exclusion criteria included those with experience limited to only being a participant in or a subject of research.

Survey information was disseminated via email distribution lists to healthcare workers, researchers and/or consumers within the participating sites and advertised on hospital noticeboards and newsletters. Up to two reminders were emailed via the same distribution lists. Snowball sampling was used by encouraging participants to forward the survey to known associates. An invitation to attend the priority-setting workshop was extended to participants who had previously contributed to the study and key stakeholders, such as know senior researchers with CCI expertise and established CCI networks from each participating site.

### Data collection

#### Surveys

Surveys were collaboratively designed for researcher and consumer respondents. Study data were collected using REDCap [39] electronic data tools hosted at Queensland Health. Multiple-choice questions, open-ended and Likert responses were developed based on existing evaluation tools (e.g. the Australian Clinical Trials Alliance (ACTA) questionnaire for evaluating CCI in research [40]; the Hennessy-Hicks Training needs analysis questionnaire [41] and CCI frameworks [38, 42]). These tools were chosen because they included questions which addressed the broad areas that were being explored by the research, and which no prior single tool had been designed to capture. The survey was divided into four distinct sections to aid navigation: section 1 - participant characteristics; section 2 - research study characteristics related to participants’ positive and challenging experiences; section 3 - overall experiences of CCI in health research, perceptions of how CCI influenced the research, and generation of priorities for improvement; and section 4 - self-reported ranking and identification of training and organisational development needs. Survey questions for both consumer and researcher participants are available in Supplementary Materials 1. The combination of demographic details; baseline research characteristics captured types and levels of CCI contributions, funding and infrastructure across research areas and methods, associated with experiences of CCI in research; evaluation of experiences, suggested barriers, and strategies for improvement; and analysis of training and organisational development needs specific to CCI in research, accommodated in-depth interpretations necessary for determining factors perceived to shape positive and meaningful CCI in research practices.

To capture the diversity of CCI in research practice between studies, section 2 of the survey asked details of CCI in research contributions across research cycle stages as defined by the National Institute for Health and Care Research (NIHR) [43] and level of influence associated with participants’ positive and challenging experiences. Questions designed to capture the extent of consumer influence on decision-making were based on the IAP2 [38] framework. The IAP2 is a widely used framework to help guide transparent approaches to community involvement activities [38]. Permission was provided to include the IAP2 matrix within the survey.

Questions in section 3 were informed by the ACTA questionnaire for evaluating CCI in research [40]. This tool includes three open-ended questions and six Likert responses on a 4-point scale. For researchers, nine tailored Likert questions were posed using the same scale. Section 4 used an adapted version of the Hennessy-Hicks Training Needs Analysis Questionnaire [41]. This questionnaire has been widely used to assess staff training needs across diverse settings and health care worker populations internationally [41, 44], and more recently to evaluate consumer-clinician partnership capabilities in health service committees in Australia [45]. Items for inclusion were based on a combination of findings from prior research that had (1) previously adapted the Hennesy-Hicks Questionnaire to determine training needs and priorities for staff undertaking research in health organisations [44], and (2) a recent qualitative study exploring researcher training needs for enhancing consumer-researcher partnerships [46]. To determine the relevance of training needs identified, 10 of the 13 questions presented to researchers were modified for a consumer audience. Participants rated the importance of each area on a 7-point scale from 1: not at all important to 7: very important, across three domain criteria (consumers) or four domain criteria (researchers).

Surveys were pilot tested for readability and flow. The consumer survey was tested by the consumer research partner (JM) and a non-research staff colleague. The researcher survey was tested by two health researchers who were not members of the research team or study participants. Minor modifications were made to the survey based on feedback, including adjustments to wording, instructions, and flow. The survey was circulated from 12th September 2024 to 6th February 2025.

#### Priority-setting workshop

Consensus meetings are commonly used to confirm priorities for change [47]. A facilitated workshop design was applied to review qualitative findings from themed experiences and suggested priorities for change and quantitative findings of top ranked training and organisational development needs from the survey, across stakeholder groups, to collaborative agree on the top priorities for change. The 2-hour workshop employed structured facilitation and group voting to support inclusive discussions. Groups were intentionally designed to include a mix of researchers and consumers, and participants given opportunities to discuss, explore concepts, and confirm details prior to voting rounds. To facilitate collaboration and manage any power imbalances between consumers and researchers, the workshop began with an open discussion of ground rules and an icebreaker to build trust and relationships within groups. All present were informed of the needs and priority suggestions identified across survey results in the form of a short presentation and the provision of infographic summaries to support understanding. The initial voting round involved participants individually adding a coloured sticker dot next to each of their top two priorities. Votes per item were tallied across groups (of min = 3; max = 5) and collectively reviewed. Items receiving zero votes were excluded from the second round of voting, which involved participants individually adding a star sticker to their top selection. Workshop facilitators moved between group discussions to ensure equal idea sharing, confirm understanding of concepts, and to collect individual votes for tallying between rounds of voting. Workshop discussions and voting procedures were facilitated by LA and KAB.

#### Consumer and community involvement

To maintain reflexivity, we regularly debriefed and documented our decisions. A consumer research partner (JM) was involved as an investigator throughout the process, from conceptualisation to analysis and dissemination, reimbursed accordingly; they were not a study participant. Consumers were invited to participate by researchers and through consumer networks. No participants received incentives for completing the online survey. Consumers who participated in the priority-setting workshop were offered free parking and remuneration.

### Analysis

The analysis employed a mixed qualitative and quantitative approach to summarise the survey responses. Descriptive statistics were used to characterise participants (Section 1) and to aggregate responses to closed-ended questions across all survey sections. Likert-scale responses, Sections 3 (scale, 1–4) and 4 (scale, 1–7), were analysed to determine the mean ratings of perceived importance and relevance of various aspects of consumer-researcher partnerships.

Qualitative content analysis [48] was applied to open-ended responses from Sections 3 and 4. Both inductive and deductive coding approaches were employed. One researcher (LA) conducted the initial coding of all responses. For reliability, a second researcher (JS or KC for researcher responses; ED, SM, or KAB for consumer responses) reviewed and validated the coding and emerging themes. Coding and thematic development were iterative and managed in Microsoft Excel. The authorship team confirmed the final themes.

Established methods from the Hennessy-Hicks tool [41] were used to interpret responses to Section 4 (training needs analysis). This involved using paired sample t-tests to calculate the difference between mean importance (domain-criteria A) and performance (domain-criteria B) scores for each item. Parametric tests are considered acceptable for use with Likert data [41, 49], and considered robust enough to examine differences in means with small sample sizes (*n* > 5) [49], unequal variances, and with moderate non-normality [49]. However, to minimise risk of type 1 errors, normality was assessed using Shapiro Wilk and visualisation tests with a moderate non-normal distribution evident for one question in each stakeholder group, and a conservative P-value (*p* < 0.01) was applied to confirm significance of findings across each sample. In addition, to test significance across multiple comparisons, a modified Bonferroni approach, using the Benjamini-Hochberg False Discovery Rate adjusted p-value calculation [50], was applied to ranked p-values. All four domain criteria from the Hennessy-Hicks tool were included to assess training needs versus organisational development needs.

#### Generating priorities for improvement

Priority lists, generated from open field responses by consumers and researchers (survey Section 3), were combined prior to theming. This involved coding of raw content, grouping codes into categories, and identifying overarching themes. Initial themes were reviewed with consumer co-author (JM) prior to refining. Refined priority themes, based on qualitative survey data, were then combined with preliminary quantitative survey findings, ranked items from the training needs analysis (survey Section 4), to develop a comprehensive understanding of priorities for improving CCI in research practices, according to diverse researcher and consumer perspectives. Integrated priorities were refined and grouped into four overarching categories (priority domains) to present at the priority-setting workshop for voting. Data triangulation in this convergent mixed-methods design [51, 52] involved confirming: (1) the validity of priority theme domains and items, i.e. consistency of suggestions across both researcher and consumer participant’s qualitative responses; and (2) deriving more nuanced priority theme domains and items from meta-synthesis of combined quantitative and qualitative survey data in the generation of priorities, prior to voting. An example of data linking for theme domain 1 is provided in Supplementary Materials 2. Preliminary analyses of qualitative and quantitative survey responses were displayed as infographics at the priority-setting workshop. Supplementary Materials 3 provides an example.

### Reflexivity statement

The post-doctoral researcher leading data collection had no prior affiliations with the participants, health service or research institutions, which helped reduce potential observer bias. However, their background as an experienced CCI researcher and dietitian shaped initial assumptions, which were balanced through team discussions and consumer input.

## Results

A total of 54 participants completed the survey (researchers: *n* = 38; consumers: *n* = 16). Table 1 provides an overview of participant characteristics. Of the 54 surveys included in the analysis, two researcher respondents did not complete section 4 therefore, only their data from Sections 1–3 have been included. Additionally, two consumer respondents did not complete Sections 3–4, and one consumer did not complete Section 4, see supplementary material 4. Participant group in illustrative quotes reported from open-field responses is indicated by a ‘C’ (consumer) or ‘R’ (researcher), e.g. C-1 or R-1, respectively.

**Table 1:** Overview of participant characteristics

Participant characteristics (*n* = 54) (survey section 1)	Consumer (*n* = 16)		Researcher (*n* = 38)	
	*n*	(%)	*n*	(%)
**Age**				
< 25 years	0	0%	0	0%
25–34 years	2	13%	8	21%
35–44 years	1	6%	13	34%
45–54 years	5	31%	10	26%
55–64 years	3	19%	7	18%
65–74 years	2	13%	0	0%
75–84 years	3	19%	0	0%
> 84 years	0	0%	0	0%
**Gender**				
Female	7	44%	33	87%
Male	9	56%	5	13%
Non-binary	0	0%	0	0%
Different term	0	0%	0	0%
Prefer not to answer	0	0%	0	0%
**Prior research-consumer partnership experience**				
One experience	3	19%	3	8%
More than one experience	13	81%	35	92%
**Years research-consumer partnership experience**				
</= 1 year	2	13%	3	8%
1-3years	6	38%	11	29%
3–5 years	5	31%	12	32%
5–10 years	1	6%	6	16%
> 10 years	2	13%	6	16%
**Received training in consumer and community involvement in research**				
Yes	12	75%	26	68%
No	4	25%	12	32%
**Consumer diversity (self-identified)**				
Chronic disability	12	57%	-	-
Born overseas	4	19%	-	-
Caregiver	4	19%	-	-
English as second language	1	5%	-	-
**Researchers lead employer**				
Public Hospital	-	-	9	59%
University	-	-	13	41%
**Researcher professional background**				
Allied Health	-	-	20	44.5%
Nursing	-	-	2	4.5%
Medical	-	-	1	2%
Research focused role (research institute)	-	-	13	29%
Research focused role (hospital based)	-	-	9	20%
*Note: Participants could select more than 1 option*				
**Researcher experience**				
</= 1 year	-	-	0	0%
1–3 years	-	-	0	0%
3–5 years	-	-	9	24%
5–10 years	-	-	9	24%
> 10 years	-	-	20	53%
**Proportion of their research involving consumers**				
Very little (< 10%)	-	-	5	13%
Some (20–40%)	-	-	10	26%
About half (50%)	-	-	5	13%
Most (60–80%)	-	-	13	34%
All (100%)	-	-	5	13%

**Survey response rate**. Across institutions, a total of 1012 research studies were active with research ethics and governance approvals in place during the recruitment period. However, response rate could not be confirmed as there was no way to determine CCI involvement in the research studies, nor the denominator sample size for calculations.

### Survey results

**Section 2.** A variety of research types and study designs were represented, with 50% of consumers and 31% of researchers reported having at least one challenging experience of CCI in research. Table 2 provides an overview of research characteristics associated with experiences. Both consumers and researchers reported positive and challenging experiences of CCI that spanned the research cycle, with consumers reporting a challenging experience most often associated with securing research funding or involvement in disseminating research. Comparatively, reports from researchers who acknowledged having a challenging CCI in research experience most often addressed the stage of deciding how to do the research or data analysis. Challenging experiences for researchers were additionally associated with remuneration payments (33% negative, 22% positive) and qualitative research designs (40% negative, 26% positive) or co-design research (30% negative, 28% positive). Positive experiences most often reported by consumers, were associated with receiving CCI in research training (16% positive, 7% negative), having dedicated consumer support workers (44% positive, 13% negative), and activities taking place at higher levels of influence (38% positive, 14% negative, at the level of collaborate). Overall, positive experiences often reported by researchers, were associated with earlier stages of the research cycle when deciding what to research 16%, securing funding 16%, or deciding how to do the research 16%, and similarly to consumers, for activities taking place at higher levels of influence on decision making (37% positive, 32% negative, at the level of collaborate). Additionally, researchers reported experiences associated with consumer levels of decision making per research cycle stage. Researchers reported that consumers did not contribute to decision making most often at the stage of securing research funding (29% of positive experiences, 67% of negative experiences). Across research respondents who identified CCI in research experiences at the collaborate level of consumer decision making, positive experiences were most often associated with research cycle stages of deciding how to do the research (32% of positive experiences [*n* = 38], 33% of negative experiences [*n* = 12]), dissemination (26% of positive experiences [*n* = 38], 17% of negative [*n* = 12]) and implementation and translation (26% of positive experiences [*n* = 38], 17% of negative [*n* = 12]).

**Table 2:** Overview of research characteristics associated with participant reported partnership experiences

Experience characteristics Participant contributors (*n* = 54) (survey section 2)	Positive		Challenging		Positive		Challenging	
	Researcher (*n* = 38)				Consumer (*n* = 16)			
	*n* (38)	(%)	*n* (12)	(%)	*n* (16)	(%)	*n* (8)	(%)
**Challenging CCI experience**								
Yes	-	-	12	31%	-	-	8	50%
No	-	-	26	68%	-	-	7	44%
did not respond	-	-	0	0%	-	-	1	6%
**Type of research**								
Clinical research	14	37%	5	42%	5	31%	4	50%
Health services research	17	45%	6	50%	7	44%	2	25%
Basic science	0	0%	0	0%	1	6%	0	0%
Public health	3	8%	0	0%	2	13%	1	13%
Not sure	4	11%	1	8%	1	6%	1	13%
**Study design / research methods** *(could select more than one option)*								
Randomised Controlled Trial	9	11%	2	10%	-	-	-	-
Qualitative	21	26%	8	40%	-	-	-	-
Co-design	23	28%	6	30%	-	-	-	-
Review	0	0%	1	5%	-	-	-	-
Cohort	8	10%	1	5%	-	-	-	-
Case study/observational study	5	6%	0	0%	-	-	-	-
Survey/chart audit	6	7%	1	5%	-	-	-	-
Epidemiological	1	1%	0	0%	-	-	-	-
Longitudinal	2	2%	1	5%	-	-	-	-
Other	6	7%	0	0%	-	-	-	-
**Research was a funded project**								
Yes	34	89%	10	83%	-	-	-	-
No	3	8%	2	17%	-	-	-	-
Unsure	1	3%	0	0%	-	-	-	-
**Project resourcing / funding to support consumer partners**								
Remuneration payments	25	22%	11	33%	13	25%	3	11%
Parking vouchers	24	21%	5	15%	4	8%	2	7%
Gift cards	14	13%	6	18%	7	14%	3	11%
Consumer received training	11	10%	0	0%	8	16%	2	7%
Researcher received training	10	9%	2	6%	8	16%	3	11%
Other resourcing	12	11%	3	9%	4	8%	6	22%
Consumer acknowledged in other ways	16	14%	6	18%	7	14%	8	30%
**Access to a dedicated consumer support officer**								
Yes	7	18%	2	17%	7	44%	1	13%
No	27	71%	8	67%	3	19%	6	75%
Unsure	4	11%	2	17%	6	38%	1	13%
**Stage of research cycle consumer partners contributed**								
Deciding what to research	34	16%	8	15%	6	15%	1	14%
Securing funding for the research	34	16%	3	6%	3	7%	2	29%
Deciding how to do it	34	16%	9	17%	8	20%	1	14%
Doing the research (data collection)	26	12%	8	15%	6	15%	1	14%
Analysis	20	9%	9	17%	4	10%	0	0%
Dissemination	22	10%	6	11%	3	7%	2	29%
Implementation & translation	22	10%	5	9%	2	5%	0	0%
Evaluation	20	9%	5	9%	9	22%	0	0%
**Consumer level of decision making** *(according to the IAP2 framework-REF)*								
INFORM	25	13%	11	21%	1	6%	2	29%
CONSULT	29	15%	10	19%	6	38%	2	29%
INVOLVE	55	29%	14	26%	3	19%	2	29%
COLLABORATE	69	37%	17	32%	6	38%	1	14%
EMPOWER	10	5%	1	2%	0	0%	0	0%

Note: Consumer levels were provided per research stage for researcher respondents, and overall for consumers respondents, per experience shared

**Section 3.** Table 3 provides an overview of participant reported experiences of involvement. Overall, consumers most frequently ranked highest that their contributions were valued, and that they gained new skills, and ranked lowest not having clarity about their role, that they were not contributing, or not having enough training to undertake their role. Researchers ranked highest that CCI allowed them to see different perspectives on the research, and lowest that CCI did not influence ethical considerations. Two open field response data generated themes of positive and challenging experiences for consumers and researchers.

**Table 3:** Overview of participant reported experiences of consumer-researcher partnerships

Overall rating of experience				
Participant contributors (survey section 3)	Consumer (*n* = 14)		Researcher (*n* = 38)	
	**n**	**%**	**n**	**%**
Excellent	7	50%	18	47%
Good	7	50%	15	39%
Neither good nor bad	0	0%	4	11%
It has been ok	0	0%	1	3%
Awful	0	0%	0	0%
**Perceptions of the impact of CCI in research**				
**Researchers (** ***n*** ** = 38)**	**Not at all**	**A little**	**Quite a lot**	**A lot**
Made the research more patient centred	5%	0%	37%	58%
Helped ensure outcomes were meaningful to patients/community	3%	5%	42%	50%
Influenced ethical considerations (e.g. informed consent, participant safety)	18%	29%	18%	34%
Helped to address practical challenges and optimise participant experience	5%	13%	42%	39%
Helped ensure documents were understandable	8%	21%	16%	55%
Helped ensure research was generalisable study population	5%	18%	37%	39%
Improved the likelihood that consumers would support the adoption of results	8%	26%	32%	34%
Allowed you to develop new skills	3%	21%	18%	58%
Allowed you to see different perspectives on the research	5%	5%	24%	66%
**Consumers (** ***n*** ** = 14)**	**Not at all**	**A little**	**Quite a lot**	**A lot**
You were clear about your role (e.g. what you can and cannot change about the project)	7%	29%	50%	14%
You were contributing to the research / project	7%	29%	50%	14%
Your contributions were valued	0%	29%	36%	36%
You had enough training / support to undertake your role	7%	21%	57%	14%
You gained new skills / knowledge that are useful	0%	14%	50%	36%
There was enough support from the team leader / research team	0%	14%	57%	29%

### Themes related to positive experiences

Four themes were identified associated with positive experiences for both consumers and researchers. Consumer themes included: (1) Valuing lived experience; (2) Effective communication, collaboration and team synergy; (3) Support for learning and contribution; and (4) Empowerment and active involvement. Researcher themes included: (1) Building relationships and working with consumers where research interests and goals align; (2) Enhancing research relevance and impact; (3) Diversity, inclusion, and representation; and (4) Infrastructure and support for CCI. Overall, most consumer responses related to *Empowerment and active involvement.* Consumers reported feeling valued and heard when they were actively involved in research processes: “Felt empowered to have my say on behalf of consumers to ensure feedback was properly recorded and interpreted” (C-5), “Satisfaction that people with lived experience are being involved in the co-design and operations of the research project leading to better outcomes for relevant consumers” (C-17). Researcher responses related most to *Enhancing research relevance and impact*. Researchers described discovering areas of importance and new ways of doing things that helped ensure research processes were accessible, outcomes relevant, and focused on what mattered most to those with the lived experience. For example, “Consumers have unique lived experience of what matters most to them and are an integral part of the research process providing insight and perspective not otherwise available” (R-15).

Ensuring that the research was relevant. We have three consumers on our working party, and they have provided advice about indicators to include in our audits as well as helping us to rank priorities and to co-design our interventions. Having consumers assist with audits has also been very helpful (R-31).

While both consumer and researcher participants provided detailed responses related to themes of positive experiences, reporting focuses on highlighting themes related to challenging experiences, which informed the prioritisation process. Table 4 provides an overview of all positive themes with illustrative quotes.

**Table 4:** Themes of experiences of CCI in research engagement that worked well

Theme	Explanation	Representative quote
**Themes of experiences where CCI engagement worked well for consumers**		
1) Valuing lived experience	Meaningful integration of lived experience in research and codesign enhances satisfaction and relevance of outcomes.	*Being involved at an extremely early concept stage. Not tacked on after it is all decided - Oh we better find a consumer the deadline is tomorrow*. (C-16)
2) Effective communication, collaboration and team synergy	Clear, inclusive communication and positive team dynamics builds respect, enhances experiences and encourages participation.	*The respectful way health professionals treat us*,* help to educate us*,* and value our comments. I really enjoy the synergy we get and the diversity*. (C-8)
3) Support for leaning and contribution	Providing support for learning and allowing consumers to try new approaches.	*Having the genuine opportunity to ‘take a shot’ and learn as I go*,* with things I have never done before - and be supported to understand them and meaningfully contribute which meant I was a valued team member.* (C-19)
4) Empowerment and active engagement	Consumers feel valued and heard when actively involved in the research process.	*Felt empowered to have my say on behalf of consumers to ensure feedback was properly recorded and interpreted.* (C-5)
**Themes of experiences where CCI engagement worked well for researchers**		
1. Building relationships and working with consumers where research interests and goals align	Matching consumer involvement opportunities to interests makes it easier to work towards a common goal.	*Matching opportunities to individual interests and skills*. (R-18) *All consumers I’ve worked with have shown enthusiasm in the research project which makes it easier to work towards a common goal*. (R-36) *Developing good relationships and shared understanding of project goals*. (R-5)
2. Enhancing research relevance and impact	Discovering areas of importance and new ways of doing things can help ensure processes are accessible and outcomes are relevant, matter most to those with the lived experience.	*Understanding their lived experience of the condition and feedback on the research process to be undertaken helped shape the project/procedure*. (R-32) *Consumers have unique lived experience of what matters most to them and are an integral part of the research process providing insight and perspective not otherwise available*. (R-15)
3. Diversity, inclusion, and representation	Structuring activities to enable diverse involvement can keep research focused and improve research quality and credibility.	*Involving a group of consumers rather than one or two people only - this means diversity of opinion and experience which helps research*,* and safeguards continuity of consumer involvement (as some consumers are not able to be involved as much as others*,* some get sick or move on*,* etc.).* (R-16) *Structuring of activities to support consumers with TBI to be engaged in the co-design workshops (e.g.*,* ensuring that there was a discrete question to be answered vs. general discussion)* (R-13) *The consumers in my projects have kept me grounded and on track. It is normally very easy to forget the whole reason for doing the research project in the first place*,* but the consumer CI always brings back the human perspective*. (R-40)
4. Infrastructure and support for engagement	Access to consumer involvement expertise and support, and funding for consumer contributions allows making time to learn from each other.	*Funding and available CCI expertise/support (i.e. expertise on the research team) to adequately renumerate consumers for their time & have the personnel to carry out meaningful consumer involvement*,* to involve consumers in more research cycle activities and have the resourcing and know how to ensure a successful experience (meaningful consumer involvement). Successful*,* meaningful consumer involvement requires a lot of resource particularly where support is needed to be involved (i.e. language*,* cognitive changes) and it is not possible to be done very well without funding to provide the support and time that is required (in my experience).* (R-29) *Open communication between consumer and researchers*,* promoting a platform for the consumer to be heard and assuring them of the value and importance of their input and perspective*. (R-23)

### Themes related to challenging experiences

Four themes were identified that contributed to challenging experiences of CCI for both consumers and researchers. Consumer themes included: (1) insufficient consumer contributions and influence; (2) power imbalances and unclear communication; (3) insufficient role clarity and unclear expectations; and (4) practical consumer involvement challenges. Researcher themes included: (1) resourcing and management; (2) practical challenges; (3) inflexible and complex processes and remuneration systems; and (4) finding, approaching and engaging consumers who are the right fit.

For consumers, *Insufficient consumer contributions and influence* characterised by a lack of consumer contributions, “There was insufficient variation of opinion by consumers” (C-1), or inadequate facilitation that can allow dominant voices to overpower others and limit genuine input into decision-making and outcomes. One consumer describes, “…sometimes the other consumers can have a predetermined agenda or something to whinge about, which can overtake the conversation, which ultimately can leave the research lacking in decent information” (C-18). *Power imbalances and unclear communication* regarding consumer reported experiences of not being given adequate explanations of jargon, acronyms or technical terms, limited their ability to contribute when they needed to “[work] through technical knowledge and acronyms” (C-15). *Insufficient role clarity and unclear expectations* related to a perceived lack of clear role explanations and expectations regarding their role, combined with a lack of regular information from researchers about study updates, which contributed to feelings of tokenism.

Not getting updates from the researchers with where the project was up to and if I was needed for further work on the projects. This made me feel like I was just a tick box that they got a consumer to read it and therefore they had consumer involvement (C-9).

For consumers, *Practical consumer involvement challenges*, captured physical, logistic, or connectivity challenges associated with the location or scheduling of activities, and personally overcoming nervousness, “I can get very nervous, and it is important to me that I contribute” (C-8).

For researchers, *Resourcing and management*, captured the challenges researchers face with finding and sourcing funding, “Getting funds to get consumer input into the study design and for preliminary co-design work” (R-42), or appropriately budgeting for and sustaining adequate funding to resource and manage consumer contributions in research budgets, “Compensation that is meaningful and not a burden to the study budget” (R-11). *Practical challenges*, related to navigating and facilitating consumer input, “Enabling consumer[s] to have the opportunity to communicate their point of view whilst staying to time in a workshop” (R-13) or “If there’s one or two very loud voices in the room this can take over the co-design agenda” (R-14). This theme also highlighted the time intensive processes needed to adequately support meaningful involvement of consumers with diverse needs, “Involving people with intellectual disabilities meaningfully, especially in governance of research” (R-22).

Genuinely supporting consumers to be involved in research takes a lot of time and is best supported through dedicated staff e.g., consumer support project officer – it’s challenging to sustain funding for these roles unless there is centralisation of effort and funding - possible but challenging (R-16)

*Inflexible and complex processes and remuneration systems*, captured researchers’ frustration with organisational policy, procedures and finance processes that were not suited to supporting diverse consumer involvement, “Navigating complex policies in [health service] to pay consumers, along with inflexible and non-transparent payment structures” (R-45).

Finance and reimbursing consumers for their time. …this feels very disjointed. The process to reimburse consumers for their time should not be this tedious. … It wastes consumers’ time, staff time, and admin time (R-19)

…the bureaucracy and red tape that you have to go through to pay consumers, particularly for co-leads/co-investigators whose time/contribution is more than just a few meetings. …all of the rubbish going on in the periphery that makes properly engaging with consumers in meaningful research so hard (R-21)

For researchers, *Finding*,* approaching and engaging consumers who are the right fit*, related to the challenges researchers face with not knowing how best to approach consumers, “For me personally it was about approaching consumers, what to say and what not to say, how best to involve them without it feeling like I am just information dumping on them” (R-40). This also captured challenges with not knowing where to find or engage the right consumer at the right time without causing undue stress, “This type of advocacy comes at a cost to the advocates. … Significant emotional support is required, and I find my clinical background helpful in understanding their experience and needs” (R-4).

**Fig. 1:**
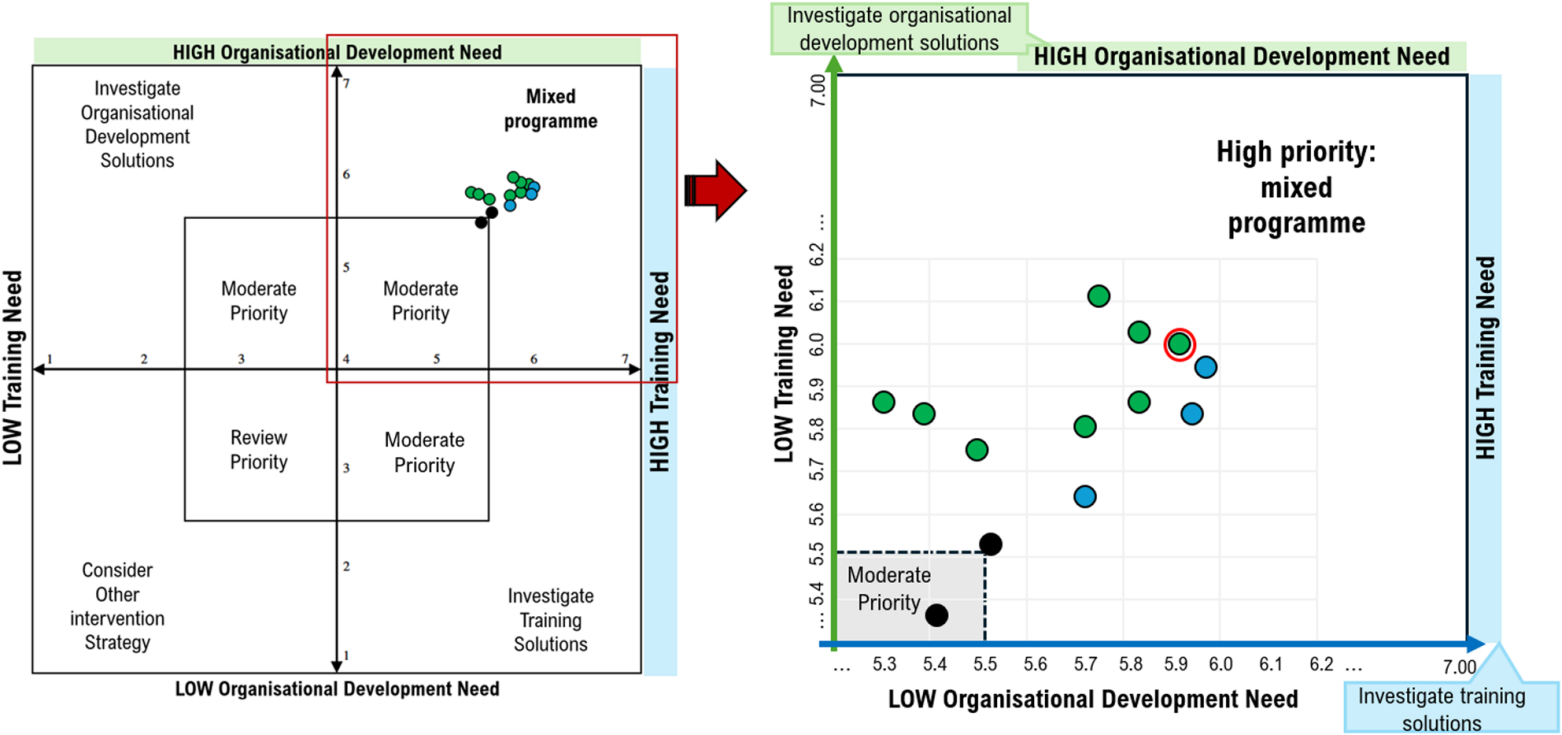
Overview of researcher ratings. Scatter plot of researcher ratings of criteria c (x-axis) and criteria d (y-axis), assessed using adapted version of the Hennessy-Hicks Training Needs Analysis Questionnaire [41]. All ratings appeared in upper right quadrant. Circled item represents item with highest combined rating. 12 items were rated high and 1 moderate for a mixed programme of both organisational development and training need. Green items rated higher for organisational development, whereas blue items rated higher for training

**Section 4.** Table 5 provides an overview of importance and performance rankings, and highest ranked organisational development and training needs. Importance was rated highly for all items. On a scale of 1 to 7, mean importance ratings for consumers ranged from 5.62 (s.d.=1.61) for teaching others how to involve consumers in research, to 6.23 for both identifying how consumers can contribute to research (s.d.=1.24) and support for consumer involvement (s.d.=1.3). A statistically significant difference was found between mean importance and mean performance scores for researcher-rated items, indicating a high training need across all items (P-values ranged from 0.003 to < 0.001, with a SD of 0.806 to 1.873). The highest training need for consumers was giving information about research to the public, while for researchers, it was identifying the right consumer for the right role. For researchers, the highest organisational development need was logistical support and resource considerations for supporting consumer involvement (mean score of 6.11 out of 7, s.d.=1.28), and the highest rated training item to enhance task performance was training in conducting research with consumers (mean score of 5.97 out of 7, s.d.=1.23). Twelve (of 13) items indicated a high priority for a mixed program of both organisational development and training, while the final item was of moderate priority (scoring below 5.5 out of 7 for both criteria), for supporting consumers to present research to the public. Eight (of 12) items rated the need for organisational development higher than training. The highest overall item across both criteria (see Fig. 1) was understanding the role of consumers in research.

**Table 5:** Overview of training and organisational development needs

Training needs analysis, Participant contributors (*n* = 49) Domain criteria description: Criteria A = importance of task Criteria B = perceived performance on task Criteria C = importance of organisational development to enhance performance Criteria D = importance of training to enhance task performance (survey section 4)	Criteria A		Criteria B		Difference in means (mean score A-B)					Criteria C		Criteria D	
	Importance		Performance		Learning development need					Organisational development		Training relevance	
	mean	rank	mean	rank	diff	rank	t	*P* value (SD) (95%CI)	BH (FDR) adjusted *p*-value	mean	rank	mean	rank
**Consumer rankings (** ***n*** ** = 13)**													
Q1. Understanding the role of consumers in the research	5.92		5.15		0.77		3.333	**0.006** (0.832)	0.018	-	-	5.54	3
Q2. Identifying how consumers can contribute to the research?	6.23	1	5.38	1	0.85		2.513	0.027 (1.214)	0.038571	-	-	5.62	2
Q3. Ensuring the right fit for the right role	6.15	2	5.08		1.08	2	3.270	**0.007** (1.188)	0.018	-	-	5.38	
Q4. Building strong relationships with the team	6.15	2	5.23	3	0.92		3.207	**0.008** (1.038)	0.018	-	-	5.23	
Q5. Conducting research as a consumer team member	6.15	2	5.08		1.08		3.092	**0.009** (1.256)	0.018	-	-	5.38	
Q6. Support for consumer involvement	6.23	1	5.38	1	0.85		2.008	0.068 (1.519)	0.068	-	-	-	-
Q7. Research integrity and ethical considerations	5.85		4.77		1.08	2	2.809	0.016 (1.382)	0.026667	-	-	5.31	
Q8. Introducing new ideas	6.00	3	5.31	2	0.69		3.323	**0.006** (0.751)	0.018	-	-	5.31	
Q9. Giving information about research to patients/the public	6.15	2	5.00		1.15	1	2.287	0.041 (1.819)	0.05125	-	-	5.77	1
Q10. Teaching others/team members how to involve consumers	5.62		4.69		0.92	3	2.009	0.068 (1.656)	0.068	-	-	5.23	
**Researcher rankings (** ***n*** ** = 36)**													
Q1. Understanding the role of consumers in the research	6.00		5.28		0.72		4.320	**< 0.001** (1.003)	**0.0010833**	6.00	3	5.92	3
Q2. Identifying how consumers can contribute to the research	6.00		5.36	3	0.64		3.872	**< 0.001** (0.990)	**0.0010833**	5.86		5.83	
Q3. Identifying the right consumer for the right role	6.03		4.72		1.31	1	6.578	**< 0.001** (1.191)	**0.0010833**	5.53		5.53	
Q8. Building strong relationships and valuing consumer involvement	6.25	2	5.67	1	0.58		4.341	**< 0.001** (0.806)	**0.0010833**	5.83		5.39	
Q10. Conducting research (with consumers)	6.28		5.33		0.94		5.759	**< 0.001** (0.984)	**0.0010833**	5.94		5.97	1
Q6. Logistic/resource considerations for supporting consumer involvement	6.11	3	4.86		1.25	2	5.275	**< 0.001** (1.422)	**0.0010833**	6.11	1	5.75	
Q9. Research integrity and ethical considerations for supporting consumers	6.36	1	5.61	2	0.75		4.170	**< 0.001** (1.079)	**0.0010833**	5.81		5.72	
Q11. (Supporting consumers to) introduce new ideas	5.86		4.81		1.06		4.842	**< 0.001** (1.308)	**0.0010833**	5.64		5.72	
Q12. (Supporting consumers to) give information about research to patients/the public	5.47		4.47		1.00		3.784	**< 0.001** (1.586)	**0.0010833**	5.36		5.42	
Q13. Teaching colleagues/students/team members how to involve consumers	5.69		4.92		0.78		3.249	**0.003** (1.436)	**0.003**	5.83		5.94	2
Q4. Applying for funding with consumer co-investigators*	5.83		4.58		1.25	2	4.005	**< 0.001** (1.873)	**0.0010833**	5.75		5.50	
Q5. Budgeting for consumer involvement*	6.06		4.92		1.14		4.254	**< 0.001** (1.606)	**0.0010833**	6.03	2	5.83	
Q7. Securing time to support consumer activities*	5.83		4.67		1.17	3	5.059	**< 0.001** (1.384)	**0.0010833**	5.86		5.31	

Notes: Starred items were presented to researchers only; listed order of questions shows items that relate to each other including alternative wording tailored for each stakeholder group. To confirm significance across multiple comparisons, a modified Bonferroni approach (Benjamini-Hochberg False Discovery Rate (BH-FDR), adjusted p-value calculation [50] was applied to adjusted p-values. All bolded results for researcher scores remained statistically significant. Bonferroni = 0.001 (consumer p-values), 0.000769 (researcher values)

Seven consumers and 16 researchers responded to the final open-field question inviting participants to offer any other feedback. Among the feedback received, responses that identified additional priorities were combined and thematically analysed along with other priority statements in preparation for the priority-setting workshop and are reflected in the final priorities identified. The five remaining responses related to aspects that worked well or were identified as additional challenges. These responses were combined and analysed with open field responses from section 3 of the survey and are captured within the themed positive and challenging experiences.

### Priority-setting workshop

**Fig. 2:**
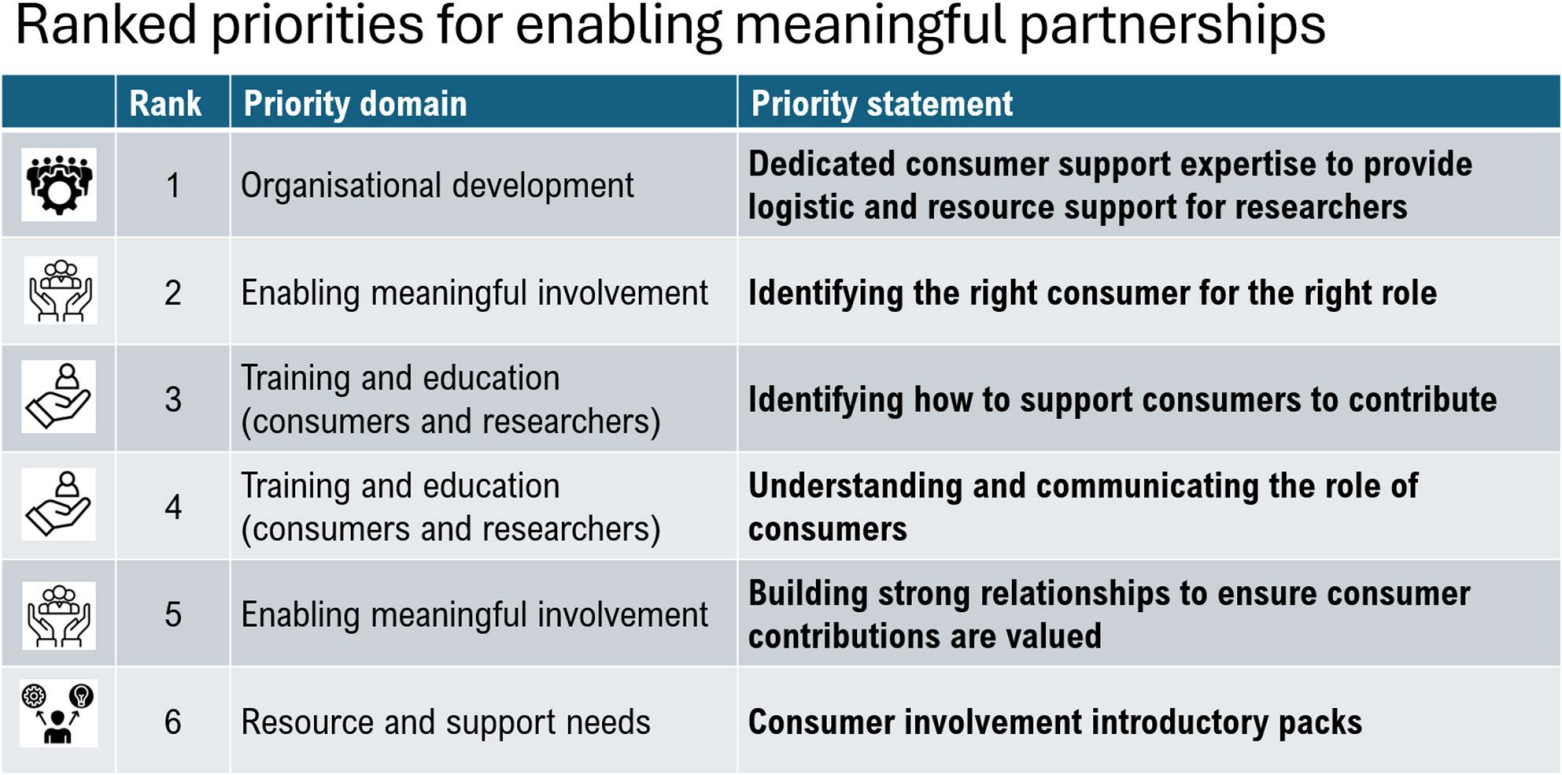
Final priority statements in ranked order

Consumers (*n* = 8) and researchers (*n* = 7) attended a priority-setting workshop (May 2025, 2 h). From the survey responses, a total of 95 priorities were suggested. Following analysis, 15 themed priority statements were identified under four domains. These included: (1) organisational development – organisational logistics and resource support need priorities (5 priority statements); (2) conducting research together - priorities for enabling meaningful involvement (4 priority statements); (3) training, educational resources or research development priorities (3 priority statements); and (4) resource and support need priorities (3 priority statements). Figure 2 provides an overview of the top six priority statements in ranked order. See Supplementary Materials 5 for further details.

### Top ranked priorities

**Priority 1.** Related to organisational development, *dedicated consumer support expertise to facilitate consumer involvement*. This priority captured the most significant needs from both consumers’ and researchers’ perspectives, for centralised dedicated support within their organisation to navigate logistical and resourcing policies and procedures. For consumers, this primarily involved accessing assistance with tracking their different partnership activities such as working between research groups, across health service directorates and research institutions and assistance with connecting and understanding new involvement opportunities. For researchers, this included dedicated consumer partnership expertise on how to adequately budget for consumer involvement or navigate relevant policies/procedures for consumer involvement.

**Priority 2. ***Identifying the right consumer for the right role*, covered an increased understanding of (1) consumer readiness acknowledging that some health care experiences can be traumatic, knowing how long to wait prior to involvement and how to adequately support involvement; (2) the value of broad lived-experience expertise; (3) how to pair lived expertise with CCI in research partnerships; and (4) consumer goals/motivations for involvement. Access to knowledge on the different ways consumers can contribute across research cycle stages or in a research service role such as grant reviews, or research committees reduced siloing of consumer networks, and ways to support consumers and researchers to connect on topics of interest were also considered.

**Priority 3. ***Identifying how to support consumers to contribute*, related to training, and captured educational needs including: (1) preparing consumers/researchers for CCI activities; (2) preparing consumers to move from research participant to active consumer; (3) research ethics associated with research partnering especially the need for supporting psychological safety; (4) research integrity/ethics for consumers; (5) consumers presenting information to the public; (6) facilitating consumer involvement; (7) mechanisms for self-evaluations of consumer-researcher partnerships.

**Priority 4. ***Understanding the role of consumers*, upskilling and training were identified for understanding: (1) the scope of consumer roles and strategies to align expectations of consumers and researchers; (2) methods of CCI and associated time requirements to support meaningful contributions; and (3) defining and supporting contributions that are not study specific such as contributions to research committees, grant review panels and research staff recruitment.

**Priority 5. ***Building strong relationships between teams to ensure consumer involvement is valued.* This priority considered strategies to describe and communicate consumer roles in each research activity by defining the purpose/intent of consumer contributions. For example, knowledge of ways to ensure consumer related activities are planned so that consumers know what to expect or supporting consumers to feel their viewpoint has been heard. Considerations of where and how consumers can contribute across the whole research process from conceptualisation to evaluation, and mechanisms for evaluating consumer involvement in research.

**Priority 6. ***Consumer involvement introductory packs* considered resource needs specific to consumer partners. This priority aimed to help consumers understand specific consumer-researcher partnership roles that they might agree to, and what these entail.

## Discussion

This research aimed to explore current CCI practices and experiences in research, and to collaboratively identify priorities and strategies for enabling meaningful CCI in research from the perspective of researchers and consumers. Findings from the survey indicate that both researchers and consumers highly value CCI in health and medical research. However, participants reported variability in how CCI is implemented across the precinct and research phases. This echoes previous studies that have identified inconsistency and fragmentation across the research cycle in how consumers are involved in research studies, impacting the quality and nature of involvement [53]. Overall, positive experiences with CCI were regularly described from early on and across multiple research cycle stages, indicating a possible shift in where and how researchers are partnering with consumers in research [8, 36]. For consumers, meaningful involvement was frequently described as feeling valued and heard by the research team. In this research, valuing lived experience encompassed providing support for learning and integrating lived experience from early on, throughout the research, concepts also consistent with prior work [36, 53, 54]. For researchers, organisational development needs were consistently rated higher than training needs, with the top-ranked item overall being understanding the role of consumers in research. Six key priorities for enhancing meaningful CCI in research were identified, pointing to organisational development, capacity building, and resource and support needs for both consumers and researchers.

### Positioning of key challenges

Challenges reported by consumers, associated with their experiences of CCI, often related to practical challenges, power imbalances, insufficient role clarity, or limited influence on the research, consistent with prior research [11, 53]. Frustrations centred around wanting facilitators to limit off-topic conversations that detracted from the time available to contribute to making meaningful change. Likewise, challenges reported by researchers were associated with resourcing, practicalities, time pressures, and onerous organisational systems and processes, which align with prior research findings [6, 11, 21, 55]. In this study, researchers additionally identified that finding and approaching consumers in the right way and at the right time to develop new collaborations was challenging. Consistent with prior research exploring the impact of CCI on research [8, 55], researchers reported that CCI helped them to see different perspectives, develop new skills, make research more patient-centred, and help ensure outcomes were meaningful to those most impacted. However, researchers in this research were divided in their opinions regarding whether CCI assisted with the ethical conduct of the research or with ensuring research was generalisable to study populations. While published literature on the impact of CCI on the ethical conduct of research is limited [21, 30, 56], for those indicating CCI did not impact ethical conduct at all in this study, it is important to note for interpretation that; (1) more than half those responding had not received any CCI training; (2) all indicated research was funded; (3) associated research fields included health services, clinical and public health research areas; and (4) research represented diverse research designs. Identifying the right consumer fit for the right role at the right time to limit any emotional impacts was a top priority for enabling meaningful CCI. Some prior research has identified that higher levels of involvement, such as collaborative decision-making, have been associated with meaningful CCI [22]. However, this research highlighted that acknowledging challenges, embracing differences, understanding lived experiences and motivations, and navigating challenges together, were the cornerstone of meaningful CCI. This finding is supported by other research in Australia, exploring the concept of meaningful collaboration between health services and consumers in healthcare quality improvement initiatives [42].

### Implications for strategy development to enable meaningful collaboration

#### Priority areas for training

Capacity building was identified as a key priority for both consumers and researchers, which affirms findings that education and training are foundational to enhancing consumer-researcher partnerships [6]. For consumers, this included upskilling and capacity building to take on different roles, in research ethics and integrity, and in different research methodologies. Researchers sought training on how to facilitate inclusive collaboration, navigate team dynamics, and ways to integrate CCI across research cycle stages and designs for maximum impact. Overwhelmingly, researchers desired training in understanding how to design, define and interpret consumer roles that are well aligned with project scopes and budgets and how to navigate role conversations with consumers to ensure mutual understanding. Additionally, researchers sought tailored guidance and skill development in how to craft role descriptions and prepare consumers to contribute to research roles outside of the research cycle, such as contributions to research committees, grant review panels, and research study staff recruitment.

#### Priority areas for organisational development

Organisational development, particularly the embedding of dedicated CCI support roles, was ranked as the top priority for improving CCI. This aligns with research findings that advocate for institutional leadership and infrastructure to normalise and sustain CCI as a core component of health research [4]. Without organisational support and resourcing, CCI efforts risk being isolated, ineffective and/or unsustainable. Also consistent with prior research, participants in this study highlighted the inconsistencies in the working of CCI policies between and across institutions [4] and research teams [57], leading to frustration, time and administrative burdens for both consumers and researchers. Priorities for change that have been less well described previously, include the role of dedicated CCI support personnel in helping consumers to keep track of different CCI commitments between and across organisations. While examples of public involvement tracking tools exist [58], consumers in this study called for CCI support roles to be extended not just to help identify and link them with suitably aligned CCI opportunities, but also to help keep them abreast of the diverse and sometimes tedious remuneration or payment systems they need to navigate. Additionally, consumers reported contributing to multiple research studies, often with different institutions in the same building, and needing help to navigate communications, expectations, and understanding of their involvement across commitments. Furthermore, dedicated CCI support roles may be an important consideration for supporting the diversification of CCI representatives, where inflexible and cumbersome organisational processes may inhibit access and involvement for consumers with unique needs such as communication or cognitive needs or differences such as aphasia, low literacy, hearing impairment, linguistic differences, or cultural needs and preferences [59].

### Future research directions

Future research should explore dedicated training options, delivered in multiple formats and including practical case-based learning opportunities, for both consumers and clinical researchers. While multiple training options exist, perhaps future research might explore or expand the evidence regarding their usefulness against the capability building needs of researchers engaging in CCI in research across different research fields of public health, clinical research, basic scientists, or health services research, and when applying different research designs. Training might also consider verifying fit for purpose, for research teams applying different research designs (e.g., the high level facilitation skills required for effective co-design, high level engagement for collaborative projects versus that of advisory groups, or other more prescriptive forms of engagement where role expectation is more clearly understood), and operating in different contexts such as clinical researchers embedded in the health service compared with researchers operating in universities or dedicated research centres. Future research should also explore strategies at the system and organisational level, required to facilitate optimal engagement, reduce cumbersome administrative processes, and provide ways to encourage and enable diversity of consumer representation in research. This should include strategies that reduce barriers such as funding structures that support access to interpreters, and multiple and flexible remuneration options. These processes should be equitable and consistent across the sector in Australia.

### Strengths and limitations

A strength of this study is its use of multiple methods, including the survey and the consensus workshop. This enabled the development of collaboratively agreed priorities that are locally relevant. This is a recognised factor for successful implementation of CCI practices [60]. Participants discussed experiences, perceived impacts of CCI, and identified training and organisational development needs associated with CCI in research. Those involved represented CCI experiences across diverse research fields, methods, research cycle stages, levels of involvement, and resourcing in Queensland, Australia, indicating potential for broad applicability of findings across a variety of research fields and study designs. The integration of the survey and consensus workshop provided a comprehensive overview of practices and perspectives, helping to clarify how to enhance CCI within the hospital and health service precinct, with wider implications for health research. The anonymous nature of data capture facilitated reporting of experiences, however, restricted ability to compare researcher and consumer responses, as there was no way to confirm where consumers or researchers were reporting on the same research activity. The recruitment of a new researcher with no prior knowledge of research studies or affiliations with researchers or consumers, to reduce bias and support objectivity in data collection and analysis, was a strength. Additionally, including perspectives from both researchers and consumers improves the comprehensiveness of the findings. However, the survey was cross-sectional, requiring participants to recall information from their past experiences. As it was not possible to determine the denominator sample size for the survey (all research studies in the precinct that involved CCI), it is not possible to determine response rate or likely non-response bias. The survey topic may have attracted participants who valued CCI in research and had more positive experiences, than those who did not respond. Furthermore, participants were recruited from one large metropolitan health precinct in Queensland, Australia which may limit the generalisability of the findings to other contexts or sites.

## Conclusions

This study identified the key training and organisational development priorities needed for implementing meaningful CCI in research. Research demonstrated increasing application of CCI across research fields, consumers reported genuine and meaningful involvement but sought clearer role definitions, and strategies for suitably pairing consumers and researchers with CCI roles. Related priorities involved enabling meaningful involvement through tools and resources that assist in matching the right consumer to the right role. This reflects the growing recognition that CCI is dynamic and involves consumers with diverse needs and preferences across diverse roles, thus, requiring tailored support. Developing structured mechanisms to support role clarity, feedback loops and self-evaluation was also emphasised. A further priority that was identified as important but less urgent was tailored resource packs for consumers, that they might use to orient themselves to different roles. Consistent national level remuneration guidelines and CCI processes and policies may be needed to address inconsistencies between and across institutions that present barriers to meaningful consumer-researcher partnerships. Implementation of the top priorities may enhance understanding of and decrease misconceptions about CCI, result in more adequately resourced CCI in research funding applications and ultimately improve experiences and outcomes for both researchers and consumers.

## Supplementary Information

Below is the link to the electronic supplementary material.

Supplementary Material 1

Supplementary Material 2

Supplementary Material 3

## Data Availability

Anonymised data that support findings are available on request from the corresponding author (LA). Raw data are not publicly available to protect participant privacy.
