# Supplementary material for: Experiences of current practice, priorities and strategies for enabling meaningful consumer and community involvement collaborations in health and medical research in Queensland, Australia
Source: Res Involv Engagem. 2026 Feb 4;12:13. doi: 10.1186/s40900-026-00847-y (PMC12870229; doi:10.1186/s40900-026-00847-y)
Supplement: Supplementary file 1 — Supplementary Material 1 [file 40900_2026_847_MOESM1_ESM.pdf]

Consumer Research Involvement Survey

Transforming person-centred care through quality consumer and community involvement in researchWhen consumers and communities join with researchers, the outcomes can improve healthcare and better meet patient needs. We don't have much information about what it is like for consumers who are part of research or what support is needed for both consumers and researchers.This survey is for:1. consumers who have been involved in research and2. researchers/clinicians who have involved consumers in research projects. Please note that participation as a research subject is not considered consumer involvement.

Thank you for your interest in this study please tell us whether you are a researcher or a consumer so we can direct you to the right the survey.

1) Are you a researcher or consumer involved in research?

◯ researcher or clinician
◯ consumer

Consumers in Research Survey

This study aims to understand how consumers have been involved in research projects at Metro North Health. This project will improve how we involve consumers in research and learn what training and support researchers and consumers need. Taking part in this study involves completing ONE online survey. Please allow up to 30 minutes to complete the survey. We recommend using a computer, however you may use a phone, or ipad. At the end of the survey, you will be invited to express your interest in completing a short interview. Please read the participant information sheet below:RBWHF-CCI_participant information sheet_4.3.24.pdf

If you have any questions about this research, please contact Lisa Anemaat: Lisa.Anemaat@health.qld.gov.au

I have read the Participant Information Sheet and consent to participate in this research and for my non-identifiable information to be used in this and future related research.

◯ Yes
◯ No

You have declined to consent and participate in this research.

Thank you for your interest in being involved.

Please provide a digital signature

____
(please sign digitally with a mouse or finger)

Do you need assistance in completing this survey form?

We can contact you to arrange assistance either over the phone or in person depending on your needs.

◯ Yes
◯ No

Name

____

Phone Number

____
(Please enter your preferred contact number, no spaces)

Please review your details.
Thank you for letting us know you need help completing this survey.

Are the details you provided correct?

Your name: [help_name]

Your phone number: [help_phone]

If these are correct please press 'Next Page' and 'Submit'. We will contact you as soon as possible, if your details are incorrect please change them above.

Definitions of terms used:
Consumer: Experience working as part of a research team conducting research.

Metro North defines a consumer as a person who has accessed or may need access to health services, including their family and carers. Consumers may represent an individual or the interests of a group based on lived experiences of healthcare they have had and according to age, gender, sexuality, cultural background or health and social needs.

Consumer involvement in research: being involved in a research project (in any way other than as a research participant).

Consumer involvement in research can occur at various stages and levels. For instance, consumers might help decide what topic to research, provide input on participant information materials, or lead workshops during data collection.

Participating as a research subject in a research project is not consumer involvement.

There are 38 questions in total (divided into 4 sections):

- Section 1 - about your involvement, information about you
- Section 2 - questions about the research you have been involved in
- Section 3 - questions about your experiences
- Section 4 - identifying training needs

Generate your unique code (using the formula below):
• First and last letter of the place you were born in (eg. Ayr = AR)
• The first initials of your name (eg. John Smith = JS)
• The month you were born as two numerals (eg. January = 01)
• Example: Unique code = ARJS01

(This code allows us to anonymously compare your experiences over time)

____

SECTION 1. Information about you (7 Questions)

Q1. Please select your age.

- ◯ 18 - 19 years
- ◯ 20 - 24 years
- ◯ 25 - 34 years
- ◯ 35 - 44 years
- ◯ 45 - 54 years
- ◯ 55 - 64 years
- ◯ 65 - 74 years
- ◯ 75 - 84 years
- ◯ 85 years and over

Q2. How do you describe your gender?

Gender refers to current gender, which may be different to sex recorded at birth and may be different to what is indicated on legal documents.

- ◯ Man or male
- ◯ Woman or female
- ◯ Non-binary
- ◯ I use a different term (please specify)
- ◯ Prefer not to answer

I use a different term to describe my gender (please specify): ______________________________

Q3. Postcode where I live: ______________________________

Q4. Do you identify with any of the following groups:

- ☐ I am of Aboriginal and/or Torres Strait Islander origin
- ☐ I was not born in Australia
- ☐ English is not my first language
- ☐ I am a caregiver of someone I live with
- ☐ I have a chronic disability/condition
- ☐ LGBTIQ+
- ☐ Other diversity
- ☐ Not applicable for me

(select all that apply)

Country of origin: ______________________________

Other diversity: ______________________________

Q5. Previous experience as a consumer member of the research team:

- ◯ I have one experience being a consumer on a research project.
- ◯ I have had many experiences being a consumer on research projects.

(select one)

Q6. Years experience contributing to research as a consumer (please respond in years.months)
If you are not sure, please give your best guess

For example: 2 years and 2 months = 2.2

____
(Please respond: [years.months]: e.g. 2.2)

Q7. Have you received training on how to engage as a consumer in research?

- ◯ Yes
- ◯ No

SECTION 2. (14 Questions)

This section asks questions about your experiences being involved in research. We want to hear about experiences that worked well and those that didn't work well for you.

The following questions are asked twice:

1. Firstly, please consider what made an experience work well.

2. Secondly, think about where things could be improved, what didn't work so well.

For the next questions, reflect on all your experiences as a consumer in health research.

Q1. Have you had an experience where things worked well?

◯ Yes
◯ No

Q2.- Please briefly describe the project you were involved in that worked well, use 1 or 2 sentences:

(Describe the research methods that were used or the type of study, e.g: a clinical trial, or a co-design project, or the research used interviews and focus groups)

____

______

Select the type of research you were involved in: (select one)

◯ Basic science research
◯ Clinical research
◯ Health service research
◯ Public health research
◯ Not sure

Q4. Where was the research conducted:

STARS = Surgical, Treatment and Rehabilitation Services
RBWH = Royal Brisbane and Women's Hospital
HBI = Herston Biofabrication Institute
JTI = Jamieson Trauma Institute
QARC = Queensland Aphasia Research Centre
RECOVER = RECOVER Injury Research Centre

☐ In a hospital (e.g. affiliated with STARS or RBWH)
☐ In a laboratory (e.g. affiliated with HBI)
☐ In the community / at a university (e.g. affiliated with JTI or university partner)
☐ Research was affiliated QARC, UQ
☐ Research was affiliated with RECOVER, UQ
☐ Other
☐ Not sure
(select any that apply)

Please specify:

____

For your research experience that worked well.

Q5. What payment or support did you receive as a consumer on the research project you described?

	yes	no	not sure
a. There was a dedicated consumer support officer	○	○	○
b. Payments (paid into my bank account)	○	○	○
c. Reimbursement for parking	○	○	○
d. Gift card provided	○	○	○
e. Training was provided for consumers	○	○	○
f. Training was provided for researchers in how to support consumer involvement	○	○	○
g. There was other funding/resourcing to support consumer involvement	○	○	○
h. Consumers were acknowledged in other ways	○	○	○

Please describe other funding/resourcing offered: ________________________________

Please describe how consumers were acknowledged: ________________________________

For your research experience that worked well.

Q6. How were you involved as a consumer in this research project:

	yes	no	planned (research is still underway)	not sure
a. Deciding what to research	◯	◯	◯	◯
b. Securing funding for the research	◯	◯	◯	◯
c. Deciding how to do it (designing the research)	◯	◯	◯	◯
d. Doing the research (data collection)	◯	◯	◯	◯
e. Analysing data (making sense of the findings)	◯	◯	◯	◯
f. Dissemination (letting people know the results)	◯	◯	◯	◯
g. Implementation (getting the findings into practice)	◯	◯	◯	◯
h. Evaluation (what could be improved / how could the research be used)	◯	◯	◯	◯

______

Which group of activities best describes your level of involvement as a consumer in the research project that worked well for you?

INFORM CONSULT INVOLVE COLLABORATE EMPOWER
e.g. hearing about the research/ attending training or a conference

e.g. providing feedback about a research document or process e.g. attending a meeting with research team to decide how to do a task e.g. member of the research team making decisions about the research e.g. research team doing what community group has decided on

◯ INFORM ◯ CONSULT ◯ INVOLVE ◯ COLLABORATE ◯ EMPOWER

For the next set of questions, reflect again on all your experiences as a consumer in health research.

Q1. Have you had an experience that didn't work well for you (i.e., could have been better)?

◯ Yes
◯ No

Q2. Please describe the research project you were involved in that didn't work well, use 1-2 sentences:

(Describe the research methods that were used or type of study: for example: a clinical trial, or a co-design project, or the research used interviews and focus groups)

____

______

Select the type of research you were involved in:

◯ Basic science research
◯ Clinical research
◯ Health service research
◯ Public health research
◯ Not sure

Q4. Where was the research conducted:

STARS = Surgical, Treatment and Rehabilitation Services
RBWH = Royal Brisbane and Women's Hospital
HBI = Herston Biofabrication Institute
JTI = Jamieson Trauma Institute
QARC = Queensland Aphasia Research Centre
RECOVER = RECOVER Injury Research Centre

☐ In a hospital (e.g. affiliated with STARS or RBWH)
☐ In a laboratory (e.g. affiliated with HBI)
☐ In the community / at a university (e.g. affiliated with JTI or university partner)
☐ Research was affiliated QARC, UQ
☐ Research was affiliated with RECOVER, UQ
☐ Other
☐ Not sure
(select any that apply)

Please specify:

____

For your research experience that didn't work well (could have been better).

Q5. Did you receive any payment or support for your contributions as a consumer to the research project you described?

	yes	no	not sure
a. There was a dedicated consumer support officer	○	○	○
b. Payments (paid into my bank account)	○	○	○
c. Reimbursement for parking	○	○	○
d. Gift card acknowledgement	○	○	○
e. Training was provided for consumers	○	○	○
f. Training was provided for researchers in how to support consumer involvement	○	○	○
g. There was other funding/resourcing to support consumer involvement	○	○	○
h. Consumers were acknowledged in other ways	○	○	○

Please describe other funding/resourcing offered: __________________________________

Please describe how consumers were acknowledged: __________________________________

For your research experience that didn't work well.

Q6. How were you involved as a consumer in this research project:

	yes	no	planned (research is still underway)	not sure
a. Deciding what to research	◯	◯	◯	◯
b. Securing funding for the research	◯	◯	◯	◯
c. Deciding how to do it (designing the research)	◯	◯	◯	◯
d. Doing the research (data collection)	◯	◯	◯	◯
e. Analysis (making sense of the findings)	◯	◯	◯	◯
f. Dissemination (letting people know the results)	◯	◯	◯	◯
g. Implementation (getting the findings into practice)	◯	◯	◯	◯
h. Evaluation (what could be improved / how could the research be used)	◯	◯	◯	◯

Which group of activities best describes your level of involvement as a consumer in the research project that didn't work well?

INFORM INFORM INVOLVE COLLABORATE EMPOWER
e.g.: hearing about the research/ attending training or a conference e.g.: providing feedback about a research document or process e.g.: attending a meeting with research team to decide how to do a task e.g.: member of the research team making decisions about the research e.g.: research team doing what community group has decided on

◯ INFORM ◯ CONSULT ◯ INVOLVE ◯ COLLABORATE ◯ EMPOWER

SECTION 3. Questions about your overall experiences as a consumer involved in health research (5 Questions)

(questions based on ACTA toolkit resources)

Q1. Overall, how would you rate your experiences of being involved as a consumer in research?

- ◯ Excellent
- ◯ Good
- ◯ Neither good nor bad
- ◯ It has been ok
- ◯ Awful

Q2. Describe one thing that worked well for you as a consumer involved in research:

____

Q3. Describe one thing you have found challenging being involved as a consumer in research:

____

Q4. If you could improve the experience for consumers involved in research, what would be your top priority?:

____

Q5. Overall, in the research you've been involved with do you feel....

	Not at all	A little	Quite a lot	A lot
You were clear about what your role was (including what you can and cannot change about the project)?	○	○	○	○
You were making a contribution to the research / project?	○	○	○	○
Your contributions were valued?	○	○	○	○
You had enough training / support to undertake your role?	○	○	○	○
You gained new skills / knowledge that are useful	○	○	○	○
There was enough support from the team leader / research team	○	○	○	○

SECTION 4. Identifying consumer's training needs (12 Questions).
If you are completing this survey on your mobile phone device, please turn your mobile on the side for this section.
These questions have been adapted from the Hennessy-Hicks Training Needs Analysis Questionnaire.

This is the last section of the survey.

Q1. Understanding the role of consumers in the research

Rank the areas below for importance to you (1 = not at all, 7 = very)

	1	2	3	4	5	6	7
How important is this activity to the successful performance of your role as a consumer team member?	○	○	○	○	○	○	○
How well do you consider that you currently perform this activity?	○	○	○	○	○	○	○
How important is the need for training in this area?	○	○	○	○	○	○	○

Q2. Identifying how consumers can contribute to the research

Rank the areas below for importance to you (1 = not at all, 7 = very)

	1	2	3	4	5	6	7
How important is this activity to the successful performance of your role as a consumer team member?	○	○	○	○	○	○	○
How well do you consider that you currently perform this activity?	○	○	○	○	○	○	○
How important is the need for training in this area?	○	○	○	○	○	○	○

Q3. Ensuring the right fit for the right role

Rank the areas below for importance to you (1 = not at all, 7 = very)

	1	2	3	4	5	6	7
How important is this activity to the successful performance of your role as a consumer team member?	○	○	○	○	○	○	○

How well do you consider that you currently perform this activity?	○	○	○	○	○	○	○
How important is the need for training in this area?	○	○	○	○	○	○	○

Q4. Building strong relationships with the team

Rank the areas below for importance to you (1 = not at all, 7 = very)

	1	2	3	4	5	6	7
How important is this activity to the successful performance of your role as a consumer team member?	○	○	○	○	○	○	○
How well do you consider that you currently perform this activity?	○	○	○	○	○	○	○
How important is the need for training in this area?	○	○	○	○	○	○	○

Q5. Conducting research as a consumer team member

Rank the areas below for importance to you (1 = not at all, 7 = very)

	1	2	3	4	5	6	7
How important is this activity to the successful performance of your role as a consumer team member?	○	○	○	○	○	○	○
How well do you consider that you currently perform this activity?	○	○	○	○	○	○	○
How important is the need for training in this area?	○	○	○	○	○	○	○

Q6. Support for consumer involvement

Rank the areas below for importance to you (1 = not at all, 7 = very)

	1	2	3	4	5	6	7
How important is this activity to being meaningfully (successfully) involved as a consumer?	○	○	○	○	○	○	○
How important is the need for training in this area?	○	○	○	○	○	○	○

Q7. Research integrity and ethical considerations

Rank the areas below for importance to you (1 = not at all, 7 = very)

	1	2	3	4	5	6	7
How important is research integrity and ethical considerations to you as a consumer team member?	○	○	○	○	○	○	○
How well do you consider that you currently contribute to this activity as a consumer team member?	○	○	○	○	○	○	○
How important is the need for training in this area?	○	○	○	○	○	○	○

Q8. Introducing new ideas

Rank the areas below for importance to you (1 = not at all, 7 = very)

	1	2	3	4	5	6	7
How important is this activity to the successful performance of your role as a consumer team member?	○	○	○	○	○	○	○
How well do you consider that you currently perform this activity?	○	○	○	○	○	○	○
How important is the need for training in this area?	○	○	○	○	○	○	○

Q9. Giving information about research to patients/the public

Rank the areas below for importance to you (1 = not at all, 7 = very)

	1	2	3	4	5	6	7
How important is this activity to the successful performance of your role as a consumer team member?	○	○	○	○	○	○	○
How well do you consider that you currently perform this activity?	○	○	○	○	○	○	○
How important is the need for training in this area?	○	○	○	○	○	○	○

Q10. Teaching others/team members how to do involve consumers

Rank the areas below for importance to you (1 = not at all, 7 = very)

	1	2	3	4	5	6	7

How important is this activity to the successful performance of your role as a consumer team member?	○	○	○	○	○	○	○
How well do you consider that you currently perform this activity?	○	○	○	○	○	○	○
How important is the need for training in this area?	○	○	○	○	○	○	○

Final two questions

Q11 - Do you have any other comments or suggestions? __________________________________

Q12 - Would you be interested in sharing more about your experiences in a short interview? ○ Yes ○ No

Thank you for your interest in an interview about your experiences as a consumer. We will contact you to provide more information about the interview.

Please provide your details.

(your contact details will be stored separately from your survey to ensure your responses are not linked to you)

Name ___________________________

Email address ___________________________

Phone ___________________________

Researchers involving Consumers Survey

This study aims to understand how consumers have been involved in research projects at Metro North Health. This project will improve how we involve consumers in research and learn what training and support researchers and consumers need. Your participation involves completing ONE online survey. Please allow up to 30 minutes to complete the survey. We recommend using a computer, however you may use a phone, or ipad. At the end of the survey, you will be invited to express your interest in a short interview. Please read the participant information sheet below:RBWHF-CCI_participant information sheet_4.3.24.pdf

If you have any questions about this research, please contact Lisa Anemaat: Lisa.Anemaat@health.qld.gov.au

I have read the Participant Information Sheet and consent to participate in this research and for my non-identifiable information to be used in this and future related research.

◯ Yes
◯ No

You have declined to consent and participate in this research.

Thank you for your interest in being involved.

Please provide a digital signature

____
(please sign digitally with a mouse or finger)

Definitions of terms used:
Consumer: Experience working as part of a research team conducting research.

Metro North defines a consumer as: a person who has accessed or may need access to health services including their family and carers. Consumers may represent an individual or the interests of a group based on lived experiences of healthcare they have had and according to age, gender, sexuality, cultural background or health and social needs.

Consumer involvement in research: being involved in a research project (in any way other than as a research participant).

Consumer involvement in research can occur at various stages and levels. For instance, consumers might help decide what topic to research, provide input on participant information materials, or lead workshops during data collection.

Participating as a research subject in a research project is not consumer involvement.

There are 44 questions in total (divided into 4 sections):

- Section 1 - about your involvement, information about you
- Section 2 - questions about the research you have been involved in
- Section 3 - questions about your experiences
- Section 4 - identifying training needs

Generate your unique code (using the formula below):
• First and last letter of the place you were born in (eg. Ayr = AR)
• The first initials of your name (eg. John Smith = JS)
• The month you were born as two numerals (eg. January = 01)
• Example: Unique code = ARJS01

(This code allows us to anonymously compare your experiences over time)

____

SECTION 1. Information about you (8 Questions)

Q1. What is your discipline or role?

- ☐ Nurse
- ☐ Medical Officer
- ☐ Allied Health professional
- ☐ Engineer
- ☐ Researcher - lead employer Health Service (e.g. MNH)
- ☐ Researcher - lead employer University (e.g. UQ)
- ☐ Public Health
- ☐ Other

(please select all that apply)

Please describe your field

____

Q1a. In your current role are you affiliated with any of the following?

- ◯ STARS = Surgical, Treatment and Rehabilitation Services
- ◯ RBWH = Royal Brisbane and Women's Hospital
- ◯ HBI = Herston Biofabrication Institute
- ◯ JTI = Jamieson Trauma Institute
- ◯ QARC = Queensland Aphasia Research Centre
- ◯ RECOVER = RECOVER Injury Research Centre
- ◯ CBCI = Comprehensive Breast Cancer Institute
- ◯ HeIDI = Herston Infectious Diseases Institute

(select one)

Q2. Years experience conducting research

- ◯ 10 years

(select one)

Q3. Please select your age.

- ◯ 18 - 19 years
- ◯ 20 - 24 years
- ◯ 25 - 34 years
- ◯ 35 - 44 years
- ◯ 45 - 54 years
- ◯ 55 - 64 years
- ◯ 65 - 74 years
- ◯ 75 - 84 years
- ◯ 85 years and over

Q4. How do you describe your gender?

Gender refers to current gender, which may be different to sex recorded at birth and may be different to what is indicated on legal documents.

- ◯ Man or male
- ◯ Woman or female
- ◯ Non-binary
- ◯ I use a different term
- ◯ Prefer not to answer

(select one)

I use a different term to describe my gender (please specify):

____

Q5. Experience working with consumers in research:

- ◯ I have one experience of consumer involvement
- ◯ I have some experience involving consumers with research (2-4 prior experiences)
- ◯ I have experience with research that regularly involves consumers

(select one)

Q6. Approximately, what proportion of your research (between Feb 2021 - now) has had consumer involvement.	◯ Very little (< 10%)<br>◯ Some (20-40%)<br>◯ About half (50%)<br>◯ Most (60-80%)<br>◯ All (100%)<br>(select one)
Q7. Years experience supporting consumers to be involved in research (please respond in years.months)<br>If you are not sure, please give your best guess<br><br>For example: 2 years and 2 months = 2.2	(Please respond: [years.months]: e.g. 2.2)
Q8. Have you received training on how to support consumer involvement in research?	◯ Yes<br>◯ No

SECTION 2. (16 Questions)

This section asks questions about your experiences conducting research with consumers.

For the next questions, reflect on all your experiences as a researcher involving consumers (between February 2021 - now). We'd like to hear about what has worked well and what didn't work well.
The following questions are asked twice:

Firstly, please consider what made an experience work well.

Secondly, consider an experience that could be improved, what didn't work so well.

Q1. Between February 2021 - now have you had an experience of consumer involement that worked well?	◯ Yes<br>◯ No
Q2. What was the title of the project or please describe the nature of the research in 1 or 2 sentences.<br><br>(note any identifying information will be removed prior to analysing results)	__________________________________
Q3. What was/is your role in the research project that worked well?	◯ Lead investigator<br>◯ Principal investigator<br>◯ Site coordinator<br>◯ Research assistant<br>◯ Other<br>(select one)
Please describe your role	______________________

Select the type of research:
(select one)

◯ Basic science research
◯ Clinical research
◯ Health service research
◯ Public health research
◯ Not sure

Q5. Select any research methods used in the project?
(select any that apply)

- ☐ Randomised controlled trial (RCT)
- ☐ Qualitative (interviews / focus groups)
- ☐ Codesign methods
- ☐ Review (systematic review / meta-analysis)
- ☐ Cohort
- ☐ Case study or case series / observation
- ☐ Clinical survey / audit / chart review
- ☐ Epidemiological study
- ☐ Longitudinal study
- ☐ Other

Please describe other methods used

____

Q6. Was the research funded?

- ◯ Yes
- ◯ No
- ◯ Not sure

Q6a. How was the research funded?
Please select the type of funding then provide the granting body

- ◯ National funding ______
- ◯ State funding ______
- ◯ Local funding ______
- ◯ Not sure

For your research consumer experience that worked well.

Q7. Did the project have funding/resourcing to support consumer involvement?

	yes	no	not sure
Access to a consumer support officer	○	○	○
Renumeration payments for consumers (paid into bank account)	○	○	○
Parking vouchers	○	○	○
Gift card/s provided	○	○	○
Training for consumers involved	○	○	○
Training for researchers in how to support consumer involvement	○	○	○
There was other resourcing to support consumer involvement	○	○	○
Consumers were acknowledged in other ways	○	○	○

Please describe other funding/resourcing offered: ______________________________

Please describe how consumers were acknowledged: ______________________________

For your research experience that worked well.

Q8. How have consumers been involved in this research project:

______

	Consumers not involved	Inform	Consult	Involve	Collaborate	Empower	N/A
Deciding what to research	○	○	○	○	○	○	○
Securing funding for the research	○	○	○	○	○	○	○
Deciding how to do it	○	○	○	○	○	○	○
Doing the research (data collection)	○	○	○	○	○	○	○
Analysis	○	○	○	○	○	○	○
Dissemination	○	○	○	○	○	○	○
Implementation & translation	○	○	○	○	○	○	○
Evaluation	○	○	○	○	○	○	○

For the next set of questions, reflect again on all your experiences as a researcher who has involved consumers in projects (between February 2021 - now).

Q9. Have you had an experience of consumer involvement that didn't work well (or could have been better)?

◯ Yes
◯ No

Q10. What was the title of the project or describe the nature of the research in 1 or 2 sentences.

(note any identifying information will be removed prior to analysing results)

____

Q11. What was/is your role in the research project that didn't work well?

◯ Lead investigator
◯ Principal investigator
◯ Site coordinator
◯ Research assistant
◯ Other
(select one)

______

Select the type of research:

◯ Basic science research
◯ Clinical research
◯ Health service research
◯ Public health research
◯ Not sure

Q13. Select any research methods used in this project
(select any that apply)

☐ Randomised controlled trial (RCT)
☐ Qualitative (interviews / focus groups)
☐ Codesign methods
☐ Review (systematic review / meta-analysis)
☐ Cohort
☐ Case study or case series / observation
☐ Clinical survey / audit / chart review
☐ Epidemiological study
☐ Longitudinal study
☐ Other

Q14. Was the research funded?

◯ Yes
◯ No
◯ Not sure

Q14a. How was the research funded?
Please select the type of funding then provide the granting body

◯ National funding ______
◯ State funding ______
◯ Local funding ______
◯ Not sure

For your research experience that didn't work well (could have been better).

Q15. Did the project have funding/resourcing to support consumer involvement?

	yes	no	not sure
Access to a consumer support officer	○	○	○
Renumeration payments for consumers (paid into bank account)	○	○	○
Parking vouchers	○	○	○
Gift card/s provided	○	○	○
Training for consumers involved	○	○	○
Training for researchers in how to support consumer involvement	○	○	○
There was other funding/resourcing to support consumer involvement	○	○	○
Consumers were acknowledged in other ways	○	○	○

Please describe other funding/resourcing offered: __________________________________

Please describe how consumers were acknowledged: __________________________________

For your research consumer experience that didn't work well.

Q16. How have consumers been involved in this research project:

______

	Consumers not involved	Inform	Consult	Involve	Collaborate	Empower	N/A
Deciding what to research	○	○	○	○	○	○	○
Securing funding for the research	○	○	○	○	○	○	○
Deciding how to do it	○	○	○	○	○	○	○
Doing the research (data collection)	○	○	○	○	○	○	○
Analysis	○	○	○	○	○	○	○
Dissemination	○	○	○	○	○	○	○
Implementation & translation	○	○	○	○	○	○	○
Evaluation	○	○	○	○	○	○	○

SECTION 3. Questions about your overall experiences as a researcher involving consumers in research (5 Questions)

(questions based on ACTA toolkit resources)

Q1. Overall, how would you rate your experience having consumers involved in research?	◯ Excellent<br>◯ Good<br>◯ Neither good nor bad<br>◯ It has been ok<br>◯ Awful
Q2. Describe one thing that worked well for you involving consumers in research.	__________________________________
Q3. Describe one thing you have found challenging supporting consumer involvement in research.	__________________________________
Q4. If you were designing ways to improve experiences of consumer involvement in research, what would be your top priority?	__________________________________

Q5. Overall, in your experience do you feel consumer involvement...

	Not at all	A little	Quite a lot	A lot
made the research more patient centred?	○	○	○	○
helped ensure research outcomes were meaningful to patients/community?	○	○	○	○
influenced ethical considerations (e.g. informed consent, participant safety)?	○	○	○	○
helped to address practical challenges and optimise participant experience?	○	○	○	○
helped ensure documents were understandable to study participants?	○	○	○	○
helped ensure the research was generalisable to the population it is intended to help?	○	○	○	○
improved the likelihood that consumers would support the adoption of results?	○	○	○	○
allowed you to develop new skills?	○	○	○	○
allowed you to see different perspectives on the research?	○	○	○	○

SECTION 4. Identifying researcher's training needs to support consumer involvement in healthcare research in Metro North (13 Questions).

When responding to each of the following questions, please consider your role in the research team. If you have been involved in multiple projects, please consider what made your experiences positive or challenging when reflecting on your role.

If you are completing this survey on your mobile phone device, please turn your mobile on the side.

These questions have been adapted from the Hennessy-Hicks Training Needs Analysis Questionnaire.

This is the last section of the survey.

Q1. Understanding the role of consumers in the research

Rank the areas below for importance to you (1 = not at all, 7 = very)

	1	2	3	4	5	6	7
How important is this activity to the successful performance of your job?	○	○	○	○	○	○	○
How well do you consider that you currently perform this activity?	○	○	○	○	○	○	○
How important is organisational change to being able to perform this activity?	○	○	○	○	○	○	○
How important is the need for training in this area?	○	○	○	○	○	○	○

Q2. Identifying how consumers can contribute to the research

Rank the areas below for importance to you (1 = not at all, 7 = very)

	1	2	3	4	5	6	7
How important is this activity to the successful performance of your job?	○	○	○	○	○	○	○
How well do you consider that you currently perform this activity?	○	○	○	○	○	○	○

How important is organisational change to being able to perform this activity?	○	○	○	○	○	○	○
How important is the need for training in this area?	○	○	○	○	○	○	○

Q3. Identifying the right consumer for the right role

Rank the areas below for importance to you (1 = not at all, 7 = very)

	1	2	3	4	5	6	7
How important is this activity to the successful performance of your job?	○	○	○	○	○	○	○
How well do you consider that you currently perform this activity?	○	○	○	○	○	○	○
How important is organisational change to being able to perform this activity?	○	○	○	○	○	○	○
How important is the need for training in this area?	○	○	○	○	○	○	○

Q4. Applying for funding with consumer co-investigators

Rank the areas below for importance to you (1 = not at all, 7 = very)

	1	2	3	4	5	6	7
How important is this activity to the successful performance of your job?	○	○	○	○	○	○	○
How well do you consider that you currently perform this activity?	○	○	○	○	○	○	○
How important is organisational change to being able to perform this activity?	○	○	○	○	○	○	○
How important is the need for training in this area?	○	○	○	○	○	○	○

Q5. Budgeting for consumer involvement

Rank the areas below for importance to you (1 = not at all, 7 = very)

	1	2	3	4	5	6	7

How important is this activity to the successful performance of your job?	○	○	○	○	○	○	○
How well do you consider that you currently perform this activity?	○	○	○	○	○	○	○
How important is organisational change to being able to perform this activity?	○	○	○	○	○	○	○
How important is the need for training in this area?	○	○	○	○	○	○	○

Q6. Logistic/resource considerations for supporting consumer involvement

Rank the areas below for importance to you (1 = not at all, 7 = very)

	1	2	3	4	5	6	7
How important is this activity to the successful performance of your job?	○	○	○	○	○	○	○
How well do you consider that you currently perform this activity?	○	○	○	○	○	○	○
How important is organisational change to being able to perform this activity?	○	○	○	○	○	○	○
How important is the need for training in this area?	○	○	○	○	○	○	○

Q7. Securing time to support consumer activities

Rank the areas below for importance to you (1 = not at all, 7 = very)

	1	2	3	4	5	6	7
How important is this activity to the successful performance of your job?	○	○	○	○	○	○	○
How well do you consider that you currently perform this activity?	○	○	○	○	○	○	○
How important is organisational change to being able to perform this activity?	○	○	○	○	○	○	○
How important is the need for training in this area?	○	○	○	○	○	○	○

Q8. Building strong relationships and valuing consumer involvement

Rank the areas below for importance to you (1 = not at all, 7 = very)

	1	2	3	4	5	6	7
How important is this activity to the successful performance of your job?	○	○	○	○	○	○	○
How well do you consider that you currently perform this activity?	○	○	○	○	○	○	○
How important is organisational change to being able to perform this activity?	○	○	○	○	○	○	○
How important is the need for training in this area?	○	○	○	○	○	○	○

Q9. Research integrity and ethical considerations for supporting consumers

Rank the areas below for importance to you (1 = not at all, 7 = very)

	1	2	3	4	5	6	7
How important is this activity to the successful performance of your job?	○	○	○	○	○	○	○
How well do you consider that you currently perform this activity?	○	○	○	○	○	○	○
How important is organisational change to being able to perform this activity?	○	○	○	○	○	○	○
How important is the need for training in this area?	○	○	○	○	○	○	○

Q10. Conducting research (with consumers)

Rank the areas below for importance to you (1 = not at all, 7 = very)

	1	2	3	4	5	6	7
How important is this activity to the successful performance of your job?	○	○	○	○	○	○	○
How well do you consider that you currently perform this activity?	○	○	○	○	○	○	○
How important is organisational change to being able to perform this activity?	○	○	○	○	○	○	○
How important is the need for training in this area?	○	○	○	○	○	○	○

Q11. (Supporting consumers to) introducte new ideas

Rank the areas below for importance to you (1 = not at all, 7 = very)

	1	2	3	4	5	6	7
How important is this activity to the successful performance of your job?	○	○	○	○	○	○	○
How well do you consider that you currently perform this activity?	○	○	○	○	○	○	○
How important is organisational change to being able to perform this activity?	○	○	○	○	○	○	○
How important is the need for training in this area?	○	○	○	○	○	○	○

Q12. (Supporting consumers to) give information about research to patients/the public

Rank the areas below for importance to you (1 = not at all, 7 = very)

	1	2	3	4	5	6	7
How important is this activity to the successful performance of your job?	○	○	○	○	○	○	○
How well do you consider that you currently perform this activity?	○	○	○	○	○	○	○
How important is organisational change to being able to perform this activity?	○	○	○	○	○	○	○
How important is the need for training in this area?	○	○	○	○	○	○	○

Q13. Teaching colleagues/students/team members how to involve consumers

Rank the areas below for importance to you (1 = not at all, 7 = very)

	1	2	3	4	5	6	7
How important is this activity to the successful performance of your job?	○	○	○	○	○	○	○
How well do you consider that you currently perform this activity?	○	○	○	○	○	○	○
How important is organisational change to being able to perform this activity?	○	○	○	○	○	○	○
How important is the need for training in this area?	○	○	○	○	○	○	○

Final two questions

Q14 - Do you have any other comments or suggestions?

____

Q15 - Would you be interested in sharing more about your experiences in a short interview?

◯ Yes ◯ No

Thank you for your interest in an interview about your experiences in research consumer involvement. We will contact you to provide more information about the interview.

Please provide your details.

(your contact details will be stored separately from your survey to ensure your responses are not linked to you)

Name

____

Email address

____

Phone

____
