# Supplementary material for: Experiences of current practice, priorities and strategies for enabling meaningful consumer and community involvement collaborations in health and medical research in Queensland, Australia
Source: Res Involv Engagem. 2026 Feb 4;12:13. doi: 10.1186/s40900-026-00847-y (PMC12870229; doi:10.1186/s40900-026-00847-y)
Supplement: Supplementary file 2 — Supplementary Material 2 [file 40900_2026_847_MOESM2_ESM.pdf]

Supplementary Materials 2-5

Supplementary Table 2. Integration of qualitative and quantitative data generated in survey responses

Domain	Priority name	Contributor (participant)	Qualitative data: survey section 3<br>Example codes	Linking notes	Quantitative data: survey section 4<br>HH question rankings, linked to priority statements
**1. Organisational development priorities: logistic and resource support**	1.1 Dedicated consumer support expertise	C-8<br>R-16<br>R-19<br>R-29<br>R-31	*Access to supported/dedicated consumer involvement support for staff for all consumer involvement activities (ensure collaborations are adequately funded/supported/resourced) (R.C.13)*<br>*Ways to support consumers to track different collaboration activities between teams (R.C.15)*<br>*Ways to help consumers connect with new opportunities to contribute (C.C.4)*	The overarching priority theme reflects quantitative findings (highest importance of need for organisational development to enhance performance). Priorities captured within this theme represent the logistic and support needs captured across strategies for improvements suggested in qualitative responses.<br><br>Both consumer and researcher respondents independently suggested priorities for improving meaningful CCI in research practices associated with logistic and resource support needs. Consumer and research respondents had experience of varied research methods and from different research areas.	(R) *HH.Q6: mean importance score: criteria C, rank 1 (6.11), (OD=organisational development)<br>(R) **HH.Q6: training need: criteria A-B, rank 2 (1.25)
	1.2 Small grant schemes for CCI	C-16<br>R-1<br>R-2	*Ways to ensure research budgets include resourcing to support activities (C.C.7)*<br>*Small grant schemes available for beginner researchers to fund consumer contributions (R.C.1)*<br>*Grant funding structures to accommodate better allocation of resourcing to support consumer contributions (R.C.2)*		(R) HH.Q6: importance score: rank 1 OD (6.11)<br>(R) HH.Q6: training need: rank 2 (A-B = 1.25)
	1.3 Ensure research integrity and ethical considerations are maintained	C-1<br>C-19	*Researchers and students need to be aware of risk factors associated with conditions consumer collaborators have as part of their lived-experience (C.C.11)*<br>*Consumer / researcher training on the research ethics/ integrity considerations associated with partnering with consumers (C.C.14)*		(C) ^HH.Q9: training need: rank 2 (A-B = 1.08)<br>(R) HH.Q6: importance score: rank 1 OD (6.11)<br>(R) HH.Q6: training need: rank 2 (A-B = 1.25)
	1.4 Improved systems for costing, budgets and payments	C-16<br>R-6<br>R-19<br>R-23<br>R-27<br>R-35<br>R-45	*Improved and streamlined systems / processes for remunerating consumers (across institutions/departments) (R.C.8)*<br>*Streamlined and flexible funding options available to fund consumer contributions (R.C.12)*<br>*Ways to ensure research budgets include resourcing to support activities (C.C.7)*		(R) HH.Q6: importance score: rank 1 OD (6.11)<br>(R) HH.Q6: training need: rank 2 (A-B = 1.25)
	1.5 Policy and procedures to enable diversity of consumers	C-1<br>C-7<br>R-11<br>R-15	*Ways to increase and encourage diversity / representation of hard-to-reach groups in consumer collaborations (R.C.11)*<br>*For research to include a variety of consumer voices when seeking feedback (C.C.1)*<br>*Increased transport accessibility (e.g. taxi vouchers) and navigation options to support consumers (R.C.10)*		(R) HH.Q6: importance score: rank 1 OD (6.11)<br>(R) HH.Q6: training need: rank 2 (A-B = 1.25)

HH=Hennessy-Hicks; CCI=Consumer and Community Involvement; (C)=Consumer; (R)=researcher; R.C/C.C=Researcher or Consumer unique code identifier; OD=Organisational Development
*HH.Q6 (criteria C): How important is the need of organisational development to enhance performance on: “Understanding the role of consumers in the research”
** HH.Q6 (mean difference, criteria A [task importance] – criteria B [perceived performance on task] scores) on: “Understanding the role of consumers in the research”
^HH.Q9 (mean difference, criteria A [task importance] – criteria B [perceived performance on task] scores) on: “Research integrity and ethical considerations”

Supplementary Materials 3.

Figure 1. Example of infographics providing a summary of preliminary findings provided to participants during the consensus workshop

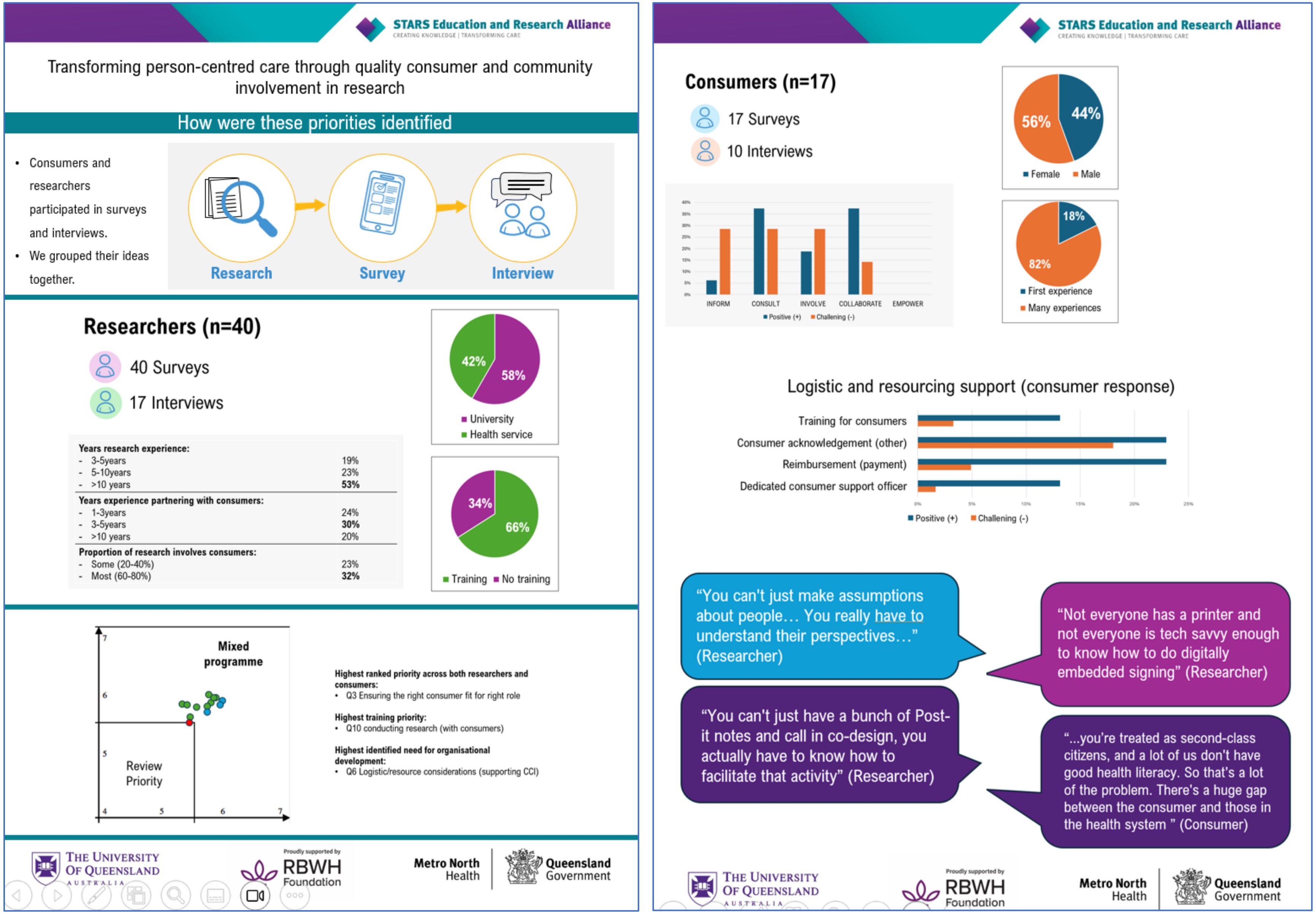

Supplementary Materials 4.

Figure 2. Overview of completed surveys included in final analysis

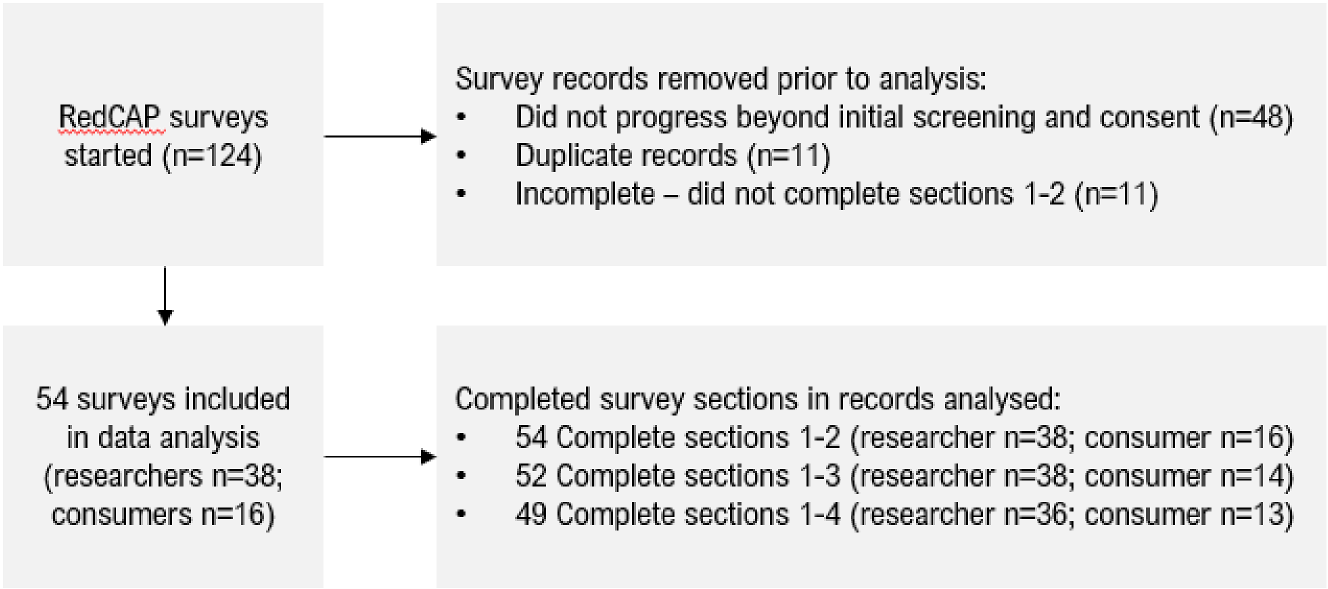

Supplementary Materials 5.

Table 1. Overview of priority domains and each priority statement including final rankings

#	Priority domain	Priority statement	Final rankings
1	**Organisational development priorities: Logistical and resource support**	1) Dedicated consumer support expertise to facilitate consumer involvement	1
		2) Small grant schemes (fund consumer contributions as co-investigators on funding applications)	
		3) Ways to ensure research integrity and ethical considerations are maintained	
		4) Improved systems for adequately costing and managing budgets for consumer involvement	
		5) Improved policy and procedures to enable diversity of consumer involvement	
2	**Conducting research together: Priorities for enabling meaningful involvement**	1) Identifying the right consumer for the right role	2
		2) Building strong relationships between teams to ensure consumers involvement is valued	5
		3) Supporting consumers to introduce new ideas	
		4) Applying for funding with consumer co-investigators	
3	**Training, educational resources, or research development priorities**	1) Identifying how to support consumers to contribute	3
		2) Understanding the role of consumers	4
		3) Budgeting for consumer involvement	
4	**Resource and support need priorities**	1) Consumer involvement introductory packs	6
		2) Peer-to-peer support networks	
		3) Securing time to support consumer activities	
