# Supplementary material for: Experiences of current practice, priorities and strategies for enabling meaningful consumer and community involvement collaborations in health and medical research in Queensland, Australia
Source: Res Involv Engagem. 2026 Feb 4;12:13. doi: 10.1186/s40900-026-00847-y (PMC12870229; doi:10.1186/s40900-026-00847-y)
Supplement: Supplementary file 3 — Supplementary Material 3 [file 40900_2026_847_MOESM3_ESM.pdf]

Guidance for Reporting Involvement of Patients and the Public version 2 long form checklist

Section and topic	Item	Reported on page No
**Section 1: Abstract of paper**		
1a: Aim	Report the aim of the study	2
1b: Methods	Describe the methods used by which patients and the public were involved	**2**
1c: Results	Report the impacts and outcomes of PPI in the study	**N/A**
1d: Conclusions	Summarise the main conclusions of the study	**2**
1e: Keywords	Include PPI, "patient and public involvement," or alternative terms as keywords	**Yes**
**Section 2: Background to paper**		
2a: Definition	Report the definition of PPI used in the study and how it links to comparable studies	**4**
2b: Theoretical underpinnings	Report the theoretical rationale and any theoretical influences relating to PPI in the study	**N/A**
2c: Concepts and theory development	Report any conceptual models or influences used in the study	**N/A**
**Section 3: Aims of paper**		
3: Aim	Report the aim of the study	**5-6**
**Section 4: Methods of paper**		
4a: Design	Provide a clear description of methods by which patients and the public were involved	**5-9**
4b: People involved	Provide a description of patients, carers, and the public involved with the PPI activity in the study	**5, 9, table 1**
4c: Stages of involvement	Report on how PPI is used at different stages of the study	**Declarations, 9, table2 1- 2**
4d: Level or nature of involvement	Report the level or nature of PPI used at various stages of the study	**Declarations, 9, tables 1-2**
**Section 5: Capture or measurement of PPI impact**		
5a: Qualitative evidence of impact	If applicable, report the methods used to qualitatively explore the impact of PPI in the study	**7-8**
5b: Quantitative evidence of impact	If applicable, report the methods used to quantitatively measure or assess the impact of PPI	**7-8**
5c: Robustness of measure	If applicable, report the rigour of the method used to capture or measure the impact of PPI	**7-8**
**Section 6: Economic assessment**		
6: Economic assessment	If applicable, report the method used for an economic assessment of PPI	**N/A**
**Section 7: Study results**		
7a: Outcomes of PPI	Report the results of PPI in the study, including both positive and negative outcomes	**9-15**
7b: Impacts of PPI	Report the positive and negative impacts that PPI has had on the research, the individuals involved (including patients and researchers), and wider impacts	**9-15**
7c: Context of PPI	Report the influence of any contextual factors that enabled or hindered the process or impact of PPI	**9-15**
7d: Process of PPI	Report the influence of any process factors, that enabled or hindered the impact of PPI	**9-15**
7ei: Theory development	Report any conceptual or theoretical development in PPI that have emerged	**N/A**
7eii: Theory development	Report testing of theoretical models, if any	**N/A**
7f: Measurement 7g: Economic assessment	If applicable, report all aspects of instrument development and testing (eg, validity, reliability, feasibility, acceptability, responsiveness, interpretability, appropriateness, precision)	**N/A**
7b: Impacts of PPI	Report any information on the costs or benefit of PPI	**N/A**
**Section 8: Discussion and conclusions**		

8a: Outcomes	Comment on how PPI influenced the study overall. Describe positive and negative effects	**15-19**
8b: Impacts	Comment on the different impacts of PPI identified in this study and how they contribute to new knowledge	**15-19**
8c: Definition	Comment on the definition of PPI used (reported in the Background section) and whether or not you would suggest any changes	**4, 15-19**
8d: Theoretical underpinnings	Comment on any way your study adds to the theoretical development of PPI	**N/A**
8e: Context	Comment on how context factors influenced PPI in the study	**15-19**
8f: Process	Comment on how process factors influenced PPI in the study	**15-19**
8g: Measurement and capture of PPI impact	If applicable, comment on how well PPI impact was evaluated or measured in the study	**N/A**
8h: Economic assessment	If applicable, discuss any aspects of the economic cost or benefit of PPI, particularly any suggestions for future economic modelling.	**N/A**
8i: Reflections/ critical perspective	Comment critically on the study, reflecting on the things that went well and those that did not, so that others can learn from this study	**N/A**

PPI=patient and public involvement

Staniszewska S, Brett J, Simera I, Seers K, Mockford C, Goodlad S, Altman DG, Moher D, Barber R, Denegri S, et al. GRIPP2 reporting checklists: tools to improve reporting of patient and public involvement in research. *BMJ (Online)*. 2017;358:j3453. doi: 10.1136/bmj.j3453
